# Supplementary material for: A fully automated, ultrasensitive luminescence cascade sensor to address hepatitis C diagnostic disparity
Source: Innovation (Camb). 2025 May 15;6(8):100952. doi: 10.1016/j.xinn.2025.100952 (PMC12347247; doi:10.1016/j.xinn.2025.100952)
Supplement: Document S1. Figures S1–S7 and Tables S1–S8 [file mmc1.pdf]

The Innovation, Volume *6*

Supplemental Information

A fully automated, ultrasensitive luminescence cascade sensor to address hepatitis C diagnostic disparity

Sungwan Kim, Adharsh Chellappaa, Juhyeon Chun, Jaebaek Lee, Joseph M. Hardie, Manoj K. Kanakasabapathy, Hemanth Kandula, Prudhvi Thirumalaraju, Gregory P. Fricker, Jenna Gustafson, Raymond T. Chung, Jorge Mera, and Hadi Shafiee

Table of Contents

Figure S1. ELISA of dAb-biotin.
Figure S2. Optimization of air volume for sample, washing buffer, and working solution injection.
Figure S3. Effect of washing buffer for the generation of bioluminescence.
Figure S4. Blocking of the channel and chamber of microfluidic cartridge.
Figure S5. Instruction sheet used for sample testing by untrained users.
Figure S6. Dose-response curve of bioluminescence intensity (RLU) from the automated assay in response to varying concentrations of serially diluted HCV-spiked samples.
Figure S7. Calculation of Cohen's κ coefficient.

Table S1. Estimated materials costs for the automated assay.
Table S2. The results obtained with the qualitative enzyme cascade-based bioluminescence system using HCV-spiked plasma samples.
Table S3. The results obtained with the qualitative enzyme cascade-based bioluminescence system using HCV-infected Cherokee Nation patient samples.
Table S4. Qualitative enzyme cascade-based bioluminescence system assessment results using HCV-spiked plasma samples.
Table S5. Qualitative conventional bioluminescence system assessment results using HCV-spiked plasma samples.
Table S6. Qualitative ELISA assessment results using HCV-spiked plasma samples.
Table S7. Qualitative qPCR assessment results using HCV-spiked plasma samples.
Table S8. Head-to-head comparison of enzyme cascade-based assay with conventional bioluminescence system, ELISA, and qPCR.

A

HCV	1	2	3	4	5	6
Core antigen	+	+	-	-	+	+
dAb-biotin	+	+	+	+	-	-
Streptavidin-HRP	+	-	+	-	+	-
Anti-IgG-HRP	-	+	-	+	-	+

B

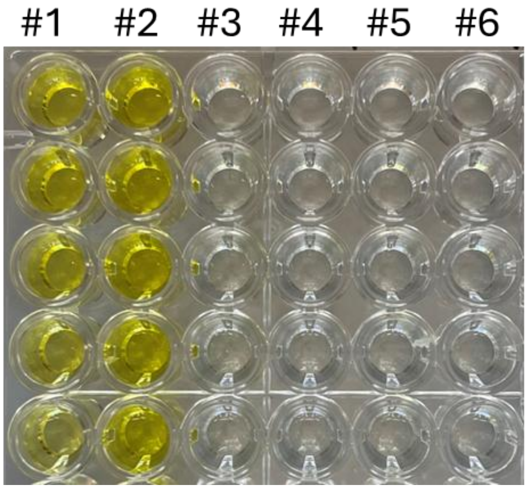

C

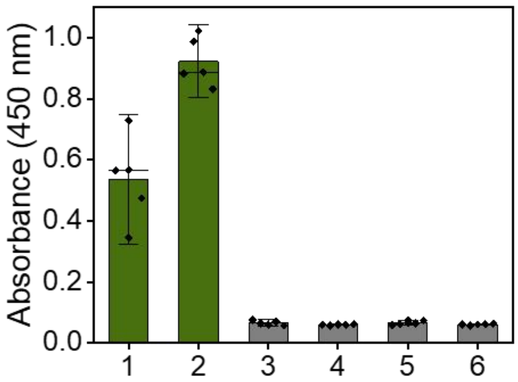

Figure S1. ELISA of dAb-biotin. (**A**) Experimental condition of ELISA. (**B**) Snapshot of the ELISA results. (**C**) ELISA analysis of dAb-biotin with antibodies targeting HCV. Bars represent mean ± SD with n = 5 technical replicates.

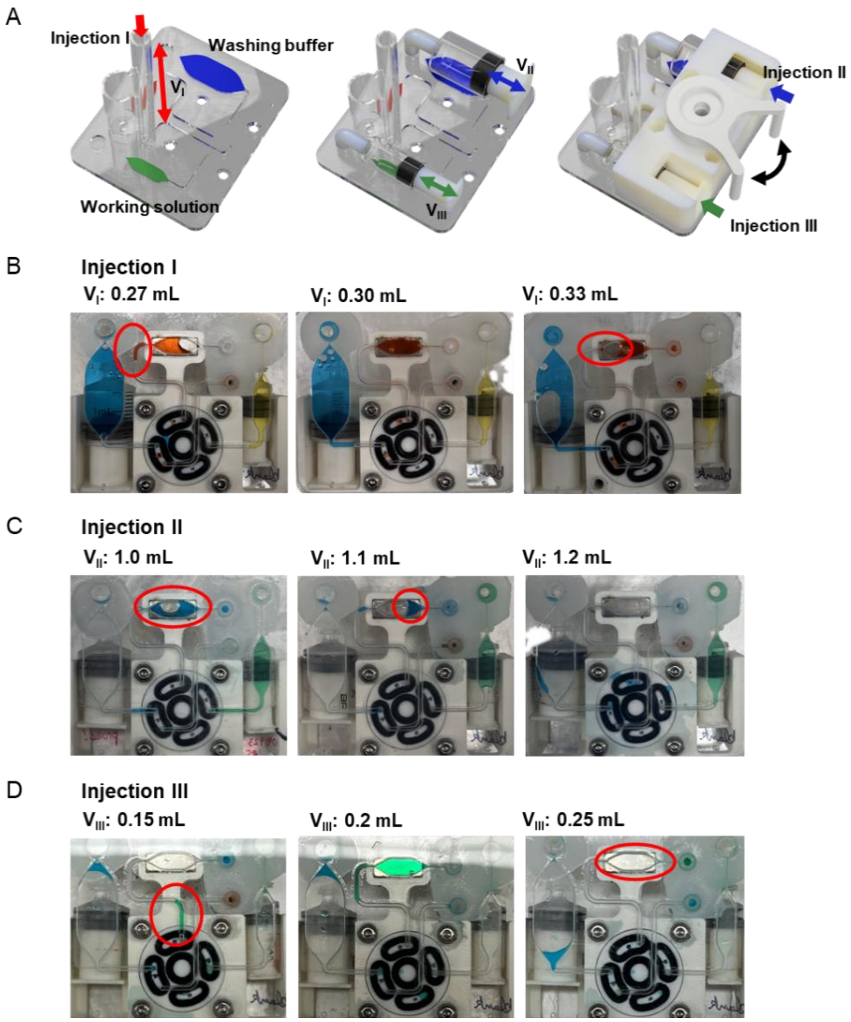

Figure S2. Optimization of air volume for sample, washing buffer, and working solution injection. (**A**) Microfluidic cartridge image. Optimization of air volume for (**B**) sample, (**C**) washing buffer, and (**D**) working solution using food dye. The colors of food dyes, red, blue, and green, correspond to sample, washing buffer, and working solution, respectively. Red circles indicate the inability of the solution to reach the appropriate position.

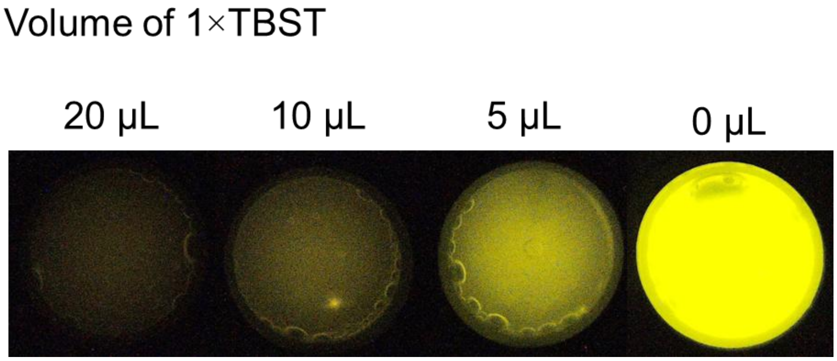

Figure S3. Effect of washing buffer for the generation of bioluminescence. The ST-GAL (10 ng) was incubated with different volume of 1×TBST (washing buffer) 0, 5, 10, 20 μL and working solution (80 μL) to generate bioluminescence.

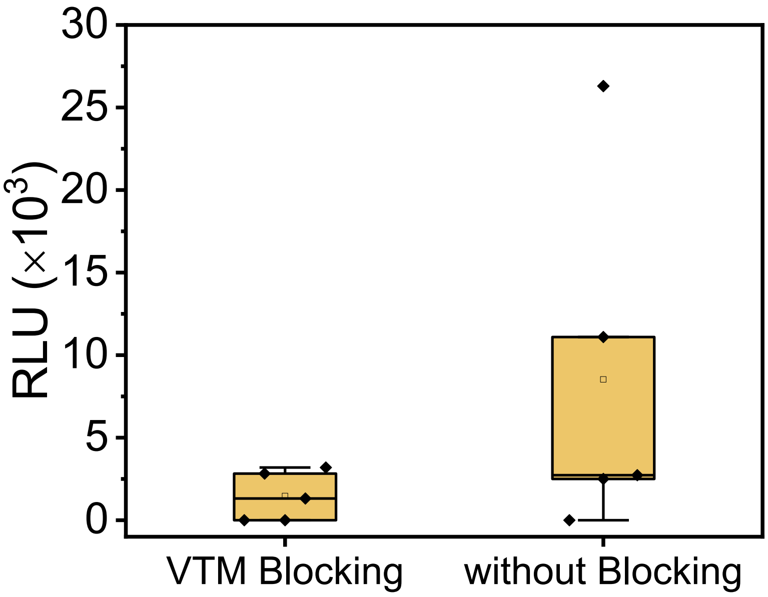

Figure S4. Blocking of the channel and chamber of microfluidic cartridge. The automated assay was performed under blank condition (using cAb-MgBeads and dAb-GAL without target protein) with or without treatment of microfluidic cartridges using VTM. Bars represent mean ± SD with n = 5 technical replicates.

User Instruction Sheet

1- Please follow these step-by-step instructions to test your sample using the extraction buffer tube (bag-1) and the detection module (bag-2). All necessary reagents are included in the provided materials.

2- Step-by-step protocol:

Step#1 Introduce the test sample into the extraction buffer tube and swirl it for 30 seconds.

Gently squeeze the tube to dispense the entire solution into the sample injection syringe.

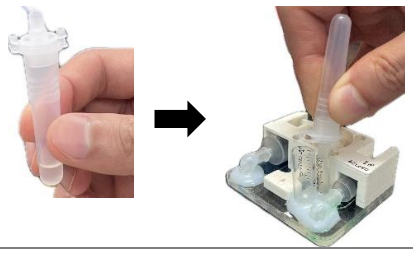

Step#2 Push the solution with injection bar.

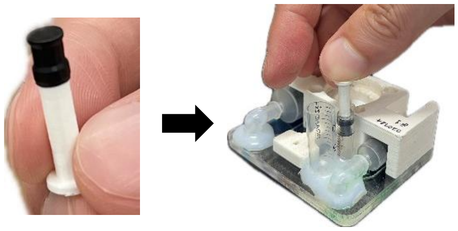

Step#3 Attach a disposable cartridge to the detection module and close the lid.

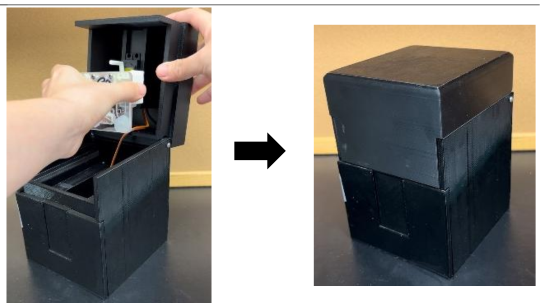

Step#4 Press the "Start" button in the smartphone app and wait for the assay result to be displayed.

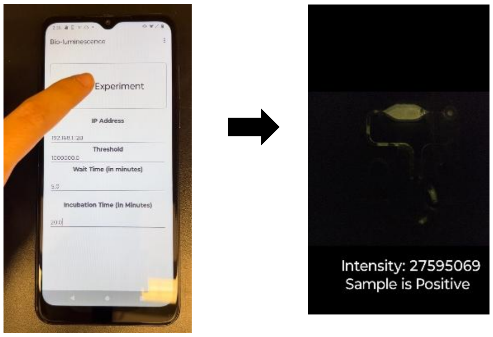

3- Dispose of the assay chamber and keep the detection module for the next experiment.

Figure S5. Instruction sheet used for sample testing by untrained users.

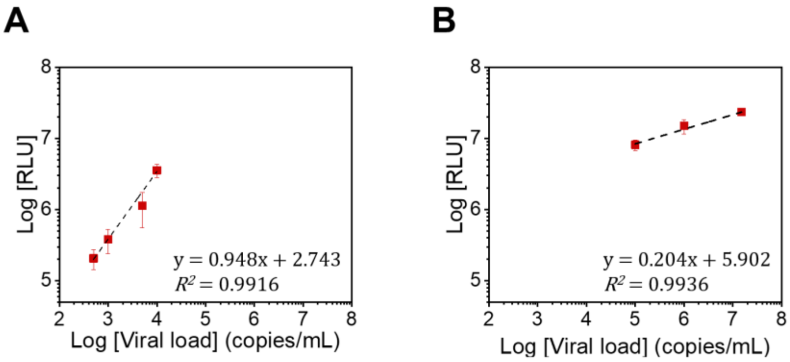

Figure S6. Dose-response curve of bioluminescence intensity (RLU) from the automated assay in response to varying concentrations of serially diluted HCV-spiked samples. (**A**) 500 to 10^4 copies/mL and (**B**) 10^5 to 10^7 copies/mL. The samples were mixed with assay materials and processed according to the protocol described in the automated assay protocol of the enzyme cascade-based bioluminescence system. Data are mean ± SD with n = 5 technical replicates.

Positive: 37
Negative: 34
False Positive: 4
False Negative: 0

A (expected True Positive): $\frac{37*71}{71} = 37$

D (expected True Negative): $\frac{34+71}{71} = 34$

B (False Positive): $\frac{4*71}{71} = 4$

C (False Negative): $\frac{0*71}{71} = 0$

$$P_0 = \frac{Number\ of\ agreements}{Total\ number\ of\ cases} = \frac{67}{71} = 0.944$$

$$P_e = \frac{(A+B)*(A+C)+(D+B)*(D+C)}{n^2} = \frac{(37+4)*(37+0)+(34+4)(34+0)}{71^2} = 0.557$$

$$\kappa = \frac{P_0 - P_e}{1 - P_e} = \frac{0.944 - 0.557}{1 - 0.557} = 0.875$$

Figure S7. Calculation of Cohen's κ coefficient.

Table S1. Estimated materials costs for the automated assay.

Item	*Cost (USD)*
Microfluidic Cartridge	
PMMA/DSA	0.001
PLA	0.119
Syringe	0.06
O-rings	0.05
cAb-MgBeads (cAb and MgBeads)	0.54
cAb	0.44
MgBeads	0.10
dAb-GAL (dAb, Site specific biotinylation and ST-GAL)	0.40
dAb	0.11
Site specific biotinylation	0.26
ST-GAL	0.03
Washing buffer	0.001
Working solution (LUGAL, Fluc, ATP, Co-enzyme A and Additives)	0.73
LUGAL	0.10
Fluc	0.50
ATP	0.11
Co-enzyme A	0.01
Additives (TCEP, EDTA, $MgSO_4 \cdot 7H_2O$ and BSA)	0.01
Bolts & Nuts	0.40
Total	2.301
Detection Module	
PLA	2.97
Arduino Nano	2.05
Raspberry Pi 4	36.38
CMOS sensor	24.99
Servo Motor	1.80
Battery	7.00
U Shape Type C Male to Female 40 Gbps Connector	1.92
Type A Male to USB 3.1 Type C Male Up Opposite U Shape Back Angled 90 Degree Charge Adapter	7.78
Jumper wires	0.175
Ball bearing 6802 RS	1.30
Bolts & Nuts	0.47
Total	86.84

Table S2. The results obtained with the qualitative enzyme cascade-based bioluminescence system using HCV-spiked plasma samples (True Positive: viral load ≥ 768 copies/mL, True Negative: viral load < 768 copies/mL, and Positive, False Positive and Negative: The results obtained by our qualitative enzyme cascade-based bioluminescence assay).

Sample #	Viral Load (copies/mL) (qPCR)	Qualitative PCR	Qualitative enzyme cascade-based bioluminescence system
1	15,000,000	True Positive	Positive
2	15,000,000	True Positive	Positive
3	15,000,000	True Positive	Positive
4	15,000,000	True Positive	Positive
5	15,000,000	True Positive	Positive
6	1,000,000	True Positive	Positive
7	1,000,000	True Positive	Positive
8	1,000,000	True Positive	Positive
9	1,000,000	True Positive	Positive
10	1,000,000	True Positive	Positive
11	100,000	True Positive	Positive
12	100,000	True Positive	Positive
13	100,000	True Positive	Positive
14	100,000	True Positive	Positive
15	100,000	True Positive	Positive
16	10,000	True Positive	Positive
17	10,000	True Positive	Positive
18	10,000	True Positive	Positive
19	10,000	True Positive	Positive
20	10,000	True Positive	Positive
21	5,000	True Positive	Positive
22	5,000	True Positive	Positive
23	5,000	True Positive	Positive
24	5,000	True Positive	Positive
25	5,000	True Positive	Positive
26	1,000	True Positive	Positive
27	1,000	True Positive	Positive
28	1,000	True Positive	Positive
29	1,000	True Positive	Positive
30	1,000	True Positive	Positive

31	500	True Negative	Negative
32	500	True Negative	Negative
33	500	True Negative	False Positive
34	500	True Negative	False Positive
35	500	True Negative	False Positive
36	350	True Negative	Negative
37	250	True Negative	Negative
38	150	True Negative	Negative
39	100	True Negative	Negative
40	80	True Negative	Negative
41	50	True Negative	Negative
42	30	True Negative	Negative
43	10	True Negative	False Positive
44	0	True Negative	Negative
45	0	True Negative	Negative
46	0	True Negative	Negative
47	0	True Negative	Negative
48	0	True Negative	Negative
49	0	True Negative	Negative
50	0	True Negative	Negative
51	0	True Negative	Negative
52	0	True Negative	Negative
53	0	True Negative	Negative
54	0	True Negative	Negative
55	0	True Negative	Negative
56	0	True Negative	Negative
57	0	True Negative	Negative
58	0	True Negative	Negative
59	0	True Negative	Negative
60	0	True Negative	Negative
61	0	True Negative	Negative
62	0	True Negative	Negative
63	0	True Negative	Negative
64	0	True Negative	Negative
65	0	True Negative	Negative
66	0	True Negative	Negative

67	0	True Negative	Negative
68	0	True Negative	Negative
69	0	True Negative	Negative
70	0	True Negative	Negative
71	0	True Negative	Negative
72	0	True Negative	Negative
73	0	True Negative	Negative
74	0	True Negative	Negative
75	0	True Negative	Negative
76	0	True Negative	Negative
77	0	True Negative	Negative
78	0	True Negative	Negative
79	0	True Negative	Negative
80	0	True Negative	Negative
81	0	True Negative	Negative
82	0	True Negative	Negative
83	0	True Negative	Negative
84	0	True Negative	Negative
85	0	True Negative	Negative
86	0	True Negative	Negative
87	0	True Negative	Negative
88	0	True Negative	Negative
89	0	True Negative	Negative
90	0	True Negative	Negative
91	0	True Negative	Negative
92	0	True Negative	Negative

Table S3. The results obtained with the qualitative enzyme cascade-based bioluminescence system using HCV-infected Cherokee Nation patient samples (True Positive: viral load ≥ 768 copies/mL, True Negative: viral load < 768 copies/mL, and Positive, False Positive, Negative and False Negative: The results obtained by our qualitative enzyme cascade-based bioluminescence assay).

Patient ID	HCV (copies/mL)	Qualitative PCR	Qualitative enzyme cascade-based bioluminescence system
HAR016	2,000	True Positive	Positive
HAR034	2,000	True Positive	False Negative
HAR032	20,000	True Positive	False Negative
HAR039	75,000	True Positive	Positive
HAR007	100,000	True Positive	Positive
HAR005	150,000	True Positive	Positive
HAR028	150,000	True Positive	Positive
HAR033	150,000	True Positive	Positive
HAR035	150,000	True Positive	Positive
HAR009	178,996	True Positive	Positive
HAR036	300,000	True Positive	Positive
HAR020	326,338	True Positive	Positive
HAR041	350,000	True Positive	Positive
HAR031	500,000	True Positive	Positive
HAR006	604,320	True Positive	Positive
HAR023	1,000,000	True Positive	Positive
HAR030	1,000,000	True Positive	Positive
HAR038	1,000,000	True Positive	Positive
HAR019	1,742,638	True Positive	Positive
HAR040	1,750,000	True Positive	Positive
HAR022	2,000,000	True Positive	Positive
HAR026	2,500,000	True Positive	Positive
HAR037	3,000,000	True Positive	Positive
HAR003	3,140,352	True Positive	Positive
HAR025	5,000,000	True Positive	Positive
HAR042	5,000,000	True Positive	Positive
HAR011	6,219,884	True Positive	Positive
HAR015	8,000,000	True Positive	Positive
HAR043	9,500,000	True Positive	Positive
HAR021	10,000,000	True Positive	Positive
HAR002	10,000,000	True Positive	Positive
HAR017	13,000,000	True Positive	Positive
HAR012	14,000,000	True Positive	Positive
HAR014	15,000,000	True Positive	Positive
HAR044	15,000,000	True Positive	Positive
HAR010	18,270,496	True Positive	Positive
HAR027	19,000,000	True Positive	Positive

Table S3. Continued.

Patient ID	HCV (copies/mL)	Qualitative PCR	Qualitative enzyme cascade-based bioluminescence system
HAR029	15	True Negative	Negative
HAR024	500	True Negative	Negative
HAR013	700	True Negative	False Positive
HAR018	718	True Negative	Negative
N1	114.25	True Negative	Negative
N2	213.5	True Negative	Negative
N3	312.75	True Negative	Negative
N4	412	True Negative	Negative
N5	511.25	True Negative	Negative
N6	50.15	True Negative	Negative
N7	85.3	True Negative	Negative
N8	120.45	True Negative	Negative
N9	155.6	True Negative	Negative
N10	190.75	True Negative	Negative
N11	225.9	True Negative	Negative
N12	261.05	True Negative	Negative
N13	296.2	True Negative	Negative
N14	331.35	True Negative	Negative
N15	366.5	True Negative	Negative
N16	401.65	True Negative	Negative
N17	436.8	True Negative	Negative
N18	471.95	True Negative	Negative
N19	507.1	True Negative	Negative
N20	542.25	True Negative	Negative
N21	39.25	True Negative	Negative
N22	63.5	True Negative	Negative
N23	87.75	True Negative	Negative
N24	112	True Negative	Negative
N25	136.25	True Negative	Negative
N26	160.5	True Negative	Negative
N27	184.75	True Negative	Negative
N28	209	True Negative	Negative
N29	510	True Negative	Negative
N30	575	True Negative	Negative

Table S4. Qualitative enzyme cascade-based bioluminescence system assessment results using HCV-spiked plasma samples (Positive and Negative: qualitative results obtained by the enzyme cascade-based bioluminescence system based on the viral load threshold of 768 copies/mL).

Positive			Negative		
Sample #	Results	Viral Load (copies/mL) (qPCR)	Sample #	Results	Viral Load (copies/mL) (qPCR)
1	Positive	15,000,000	24	Negative	700
2	Positive	13,000,000	25	Negative	500
3	Positive	1,000,000	26	Negative	350
4	Positive	900,000	27	Negative	250
5	Positive	800,000	28	Negative	150
6	Positive	700,000	29	Negative	100
7	Positive	650,000	30	Negative	80
8	Positive	500,000	31	Negative	0
9	Positive	350,000	32	Negative	0
10	Positive	250,000	33	Negative	0
11	Positive	150,000	34	Negative	0
12	Positive	100,000	35	Negative	0
13	Positive	80,000	36	Negative	0
14	Positive	60,000	37	Negative	0
15	Positive	40,000	38	Negative	0
16	Positive	20,000	39	Negative	0
17	Positive	10,000	40	Negative	0
18	Positive	7,500	41	Negative	0
19	Positive	5,000	42	Negative	0
20	Positive	2,000	43	Negative	0
21	Positive	1,500	44	Negative	0
22	Positive	1,000	45	Negative	0
23	Positive	900	46	Negative	0
-	-	-	47	Negative	0
-	-	-	48	Negative	0
-	-	-	49	Negative	0
-	-	-	50	Negative	0

Table S5. Qualitative conventional bioluminescence system assessment results using HCV-spiked plasma samples (Positive, False Negative, and Negative: qualitative results obtained by the qualitative conventional bioluminescence system based on the viral load threshold of 768 copies/mL).

Positive			Negative		
**Sample #**	**Results**	**Viral Load (copies/mL) (qPCR)**	**Sample #**	**Results**	**Viral Load (copies/mL) (qPCR)**
1	Positive	15,000,000	24	Negative	700
2	Positive	13,000,000	25	Negative	500
3	Positive	1,000,000	26	Negative	350
4	Positive	900,000	27	Negative	250
5	Positive	800,000	28	Negative	150
6	Positive	700,000	29	Negative	100
7	Positive	650,000	30	Negative	80
8	Positive	500,000	31	Negative	0
9	Positive	350,000	32	Negative	0
10	Positive	250,000	33	Negative	0
11	Positive	150,000	34	Negative	0
12	Positive	100,000	35	Negative	0
13	Positive	80,000	36	Negative	0
14	Positive	60,000	37	Negative	0
15	False Negative	40,000	38	Negative	0
16	False Negative	20,000	39	Negative	0
17	False Negative	10,000	40	Negative	0
18	False Negative	7,500	41	Negative	0
19	False Negative	5,000	42	Negative	0
20	False Negative	2,000	43	Negative	0
21	False Negative	1,500	44	Negative	0
22	False Negative	1,000	45	Negative	0
23	False Negative	900	46	Negative	0
-	-	-	47	Negative	0
-	-	-	48	Negative	0
-	-	-	49	Negative	0
-	-	-	50	Negative	0

Table S6. Qualitative ELISA assessment results using HCV-spiked plasma samples (Positive, False Negative, and Negative: qualitative results obtained by the ELISA based on the viral load threshold of 768 copies/mL).

Positive			Negative		
**Sample #**	**Results**	**Viral Load (copies/mL) (qPCR)**	**Sample #**	**Results**	**Viral Load (copies/mL) (qPCR)**
1	Positive	15,000,000	24	Negative	700
2	Positive	13,000,000	25	Negative	500
3	Positive	1,000,000	26	Negative	350
4	Positive	900,000	27	Negative	250
5	Positive	800,000	28	Negative	150
6	Positive	700,000	29	Negative	100
7	Positive	650,000	30	Negative	80
8	Positive	500,000	31	Negative	0
9	Positive	350,000	32	Negative	0
10	Positive	250,000	33	Negative	0
11	Positive	150,000	34	Negative	0
12	Positive	100,000	35	Negative	0
13	Positive	80,000	36	Negative	0
14	Positive	60,000	37	Negative	0
15	Positive	40,000	38	Negative	0
16	Positive	20,000	39	Negative	0
17	Positive	10,000	40	Negative	0
18	False Negative	7,500	41	Negative	0
19	False Negative	5,000	42	Negative	0
20	False Negative	2,000	43	Negative	0
21	False Negative	1,500	44	Negative	0
22	False Negative	1,000	45	Negative	0
23	False Negative	900	46	Negative	0
-	-	-	47	Negative	0
-	-	-	48	Negative	0
-	-	-	49	Negative	0
-	-	-	50	Negative	0

Table S7. Qualitative qPCR assessment results using HCV-spiked plasma samples (Positive and Negative: qualitative results obtained by the qPCR based on the viral load threshold of 768 copies/mL).

Positive			Negative		
**Sample #**	**Results**	**Viral Load (copies/mL) (qPCR)**	**Sample #**	**Results**	**Viral Load (copies/mL) (qPCR)**
1	Positive	15,000,000	24	Negative	700
2	Positive	13,000,000	25	Negative	500
3	Positive	1,000,000	26	Negative	350
4	Positive	900,000	27	Negative	250
5	Positive	800,000	28	Negative	150
6	Positive	700,000	29	Negative	100
7	Positive	650,000	30	Negative	80
8	Positive	500,000	31	Negative	0
9	Positive	350,000	32	Negative	0
10	Positive	250,000	33	Negative	0
11	Positive	150,000	34	Negative	0
12	Positive	100,000	35	Negative	0
13	Positive	80,000	36	Negative	0
14	Positive	60,000	37	Negative	0
15	Positive	40,000	38	Negative	0
16	Positive	20,000	39	Negative	0
17	Positive	10,000	40	Negative	0
18	Positive	7,500	41	Negative	0
19	Positive	5,000	42	Negative	0
20	Positive	2,000	43	Negative	0
21	Positive	1,500	44	Negative	0
22	Positive	1,000	45	Negative	0
23	Positive	900	46	Negative	0
-	-	-	47	Negative	0
-	-	-	48	Negative	0
-	-	-	49	Negative	0
-	-	-	50	Negative	0

Table S8. Head-to-head comparison of enzyme cascade-based assay with conventional bioluminescence system, ELISA, and qPCR.

	Enzyme cascade-based bioluminescence system	Conventional bioluminescence system	ELISA	**qPCR**
Specificity	**100%**	**100%**	**100%**	**100%**
Sensitivity	**100%**	**61%**	**74%**	**100%**
Accuracy	**100%**	**78%**	**84%**	**100%**
