# Supplementary material for: A fully automated, ultrasensitive luminescence cascade sensor to address hepatitis C diagnostic disparity
Source: Innovation (Camb). 2025 May 15;6(8):100952. doi: 10.1016/j.xinn.2025.100952 (PMC12347247; doi:10.1016/j.xinn.2025.100952)
Supplement: Document S2. Article plus supplemental information [file mmc4.pdf]

REPORT

A fully automated, ultrasensitive luminescence cascade sensor to address hepatitis C diagnostic disparity

Sungwan Kim,[1] Adharsh Chellappaa,[1] Juhyeon Chun,[1] Jaebaek Lee,[1] Joseph M. Hardie,[1] Manoj K. Kanakasabapathy,[1] Hemanth Kandula,[1] Prudhvi Thirumalaraju,[1] Gregory P. Fricker,[2] Jenna Gustafson,[2] Raymond T. Chung,[2] Jorge Mera,[3,4] and Hadi Shafiee[1,*]
*Correspondence: hshafiee@bwh.harvard.edu

Received: August 20, 2024; Accepted: May 13, 2025; Published Online: May 15, 2025; https://doi.org/10.1016/j.xinn.2025.100952

© 2025 The Author(s). Published by Elsevier Inc. on behalf of Youth Innovation Co., Ltd. This is an open access article under the CC BY license (http://creativecommons.org/licenses/by/4.0/).

GRAPHICAL ABSTRACT

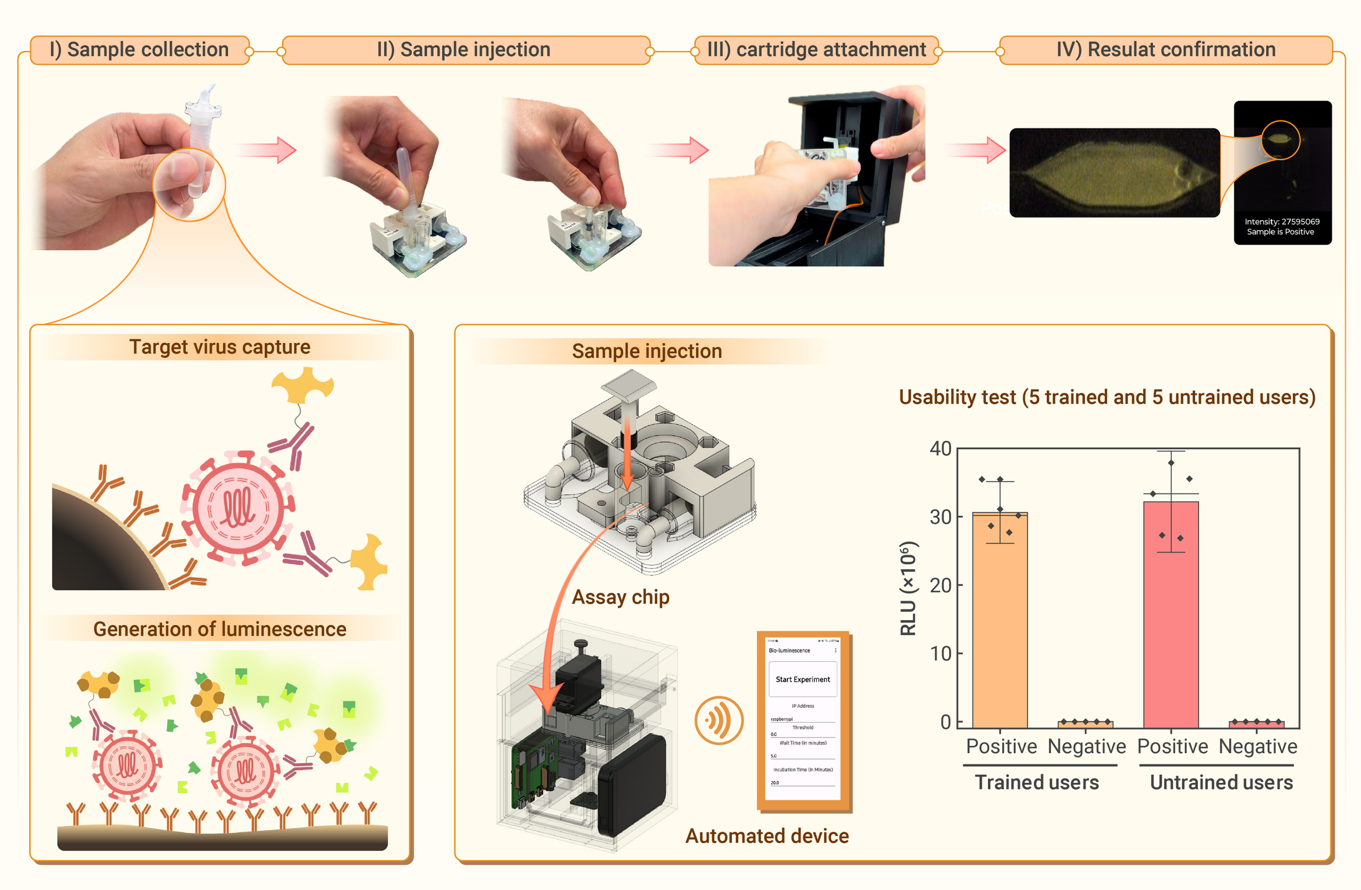

PUBLIC SUMMARY

- HCV infects 1.5 million people yearly, with American Indians and Alaska Natives most affected.
- Current two-step diagnostics are costly and slow and lead to high dropout rates among patients.
- Developed a sensitive, user-friendly, automated point-of-care antigen test based on bioluminescence imaging.
- In 71 AI/AN samples, the assay showed 97% sensitivity, 94% specificity, and 96% accuracy within 30 min.
- This technology enables early intervention and expands HCV screening in resource-limited settings.

OPEN ACCESS

REPORT

The Innovation

A fully automated, ultrasensitive luminescence cascade sensor to address hepatitis C diagnostic disparity

Sungwan Kim,[1] Adharsh Chellappaa,[1] Juhyeon Chun,[1] Jaebaek Lee,[1] Joseph M. Hardie,[1] Manoj K. Kanakasabapathy,[1] Hemanth Kandula,[1] Prudhvi Thirumalaraju,[1] Gregory P. Fricker,[2] Jenna Gustafson,[2] Raymond T. Chung,[2] Jorge Mera,[3,4] and Hadi Shafiee[1,*]

[1]Division of Engineering in Medicine, Department of Medicine, Brigham and Women's Hospital, Harvard Medical School, Boston, MA 02139, USA

[2]Liver Center, Gastrointestinal Division, Massachusetts General Hospital, Harvard Medical School, Boston, MA 02114, USA

[3]Infectious Diseases, Cherokee Nation Health Services, Tahlequah, OK 74464, USA

[4]Department of Medicine, Division of Infectious Diseases, University of New Mexico Health Sciences Center, Albuquerque, NM 87131, USA

*Correspondence: hshafiee@bwh.harvard.edu

Received: August 20, 2024; Accepted: May 13, 2025; Published Online: May 15, 2025; https://doi.org/10.1016/j.xinn.2025.100952

© 2025 The Author(s). Published by Elsevier Inc. on behalf of Youth Innovation Co., Ltd. This is an open access article under the CC BY license (http://creativecommons.org/licenses/by/4.0/).

Citation: Kim S., Chellappaa A., Chun J., et al., (2025). A fully automated, ultrasensitive luminescence cascade sensor to address hepatitis C diagnostic disparity. The Innovation **6(8)**, 100952.

Viral hepatitis poses a significant global health burden, with chronic hepatitis B and C causing about 1 million annual deaths from liver cancer and cirrhosis. Over 1.5 million new hepatitis C virus (HCV) cases arise yearly, especially among vulnerable groups like American Indians and Alaska Natives (AI/AN). Despite effective direct-acting antivirals, early HCV diagnosis remains challenging, particularly in resource-limited settings. Current two-step testing methods are costly and prone to patient dropout. Point-of-care (POC) HCV antigen (Ag) testing offers a promising early detection approach, but no US Food and Drug Administration (FDA)-approved POC test meets the sensitivity and specificity needed for low viral loads. To address this, we developed a fully automated bioluminescence-based POC assay using a cascade-based signal amplification strategy. Evaluated on 71 AI/AN samples, it showed 97% sensitivity, 94% specificity, and 96% accuracy. This technology can improve health equity by enabling accessible and reliable HCV testing for disproportionately affected populations.

INTRODUCTION

Viral hepatitis remains one of the top ten causes of global illness and death. The World Health Organization (WHO) estimates that chronic hepatitis B (hepatitis B virus [HBV]) and hepatitis C (hepatitis C virus [HCV]) infections lead to about 1 million deaths each year, primarily due to liver cancer and cirrhosis.[1] Annually, more than 1.5 million people contract hepatitis C.[1] Managing HCV effectively and promptly is particularly challenging in populations with significant health disparities. Vulnerable groups include those in resource-limited settings, rural areas, and economically disadvantaged regions; migrants; and displaced populations. American Indians and Alaska Natives (AI/AN) have an HCV infection rate of 2.9 cases per 100,000, significantly higher than the rates of African Americans (0.5 per 100,000) and non-Hispanic Whites (1.2 per 100,000). They also have higher mortality rates compared to other racial and ethnic groups.[2,3]

In response to the high incidence of HCV among AI/AN and the availability of effective direct-acting antivirals (DAAs), the Cherokee National Health Services (CNHS) implemented new testing strategies in October 2012, consistent with the 2012 CDC guidelines and the recommendations of the US Preventive Services Task Force.[2] These strategies substantially reduced patient waiting and travel times, resulting in a 5-fold increase in the percentage of AI/AN in Oklahoma tested for HCV from 2012 to 2015.[2] Nevertheless, despite these advancements, many individuals identified as HCV-antibody positive did not undergo confirmatory HCV RNA testing.[2] Despite the effectiveness of DAAs, which require simple once-daily dosing and have cure rates exceeding 95%,[4,5] challenges persist in the rapid diagnosis of active HCV infection, particularly in resource-limited settings and among disproportionately affected populations, such as AI/AN.[6] Globally, only 21% of individuals infected with HCV receive a diagnosis.[1,7–9] To meet the WHO's 2016 HCV elimination targets—an 80% reduction in new infections and a 65% reduction in mortality by 2030[10]—substantial increases in HCV screening are essential to identify and treat at least 80% of the estimated 58 million HCV-infected individuals worldwide.[11]

A major barrier to connecting HCV-infected individuals with effective DAAs is the two-step testing protocol,[12,13] which requires initial HCV antibody screening followed by HCV RNA confirmatory testing.[14] This process is expensive, time consuming, and inconvenient, leading many individuals to drop out of the care continuum before receiving appropriate treatment or confirmatory testing.[7,15,16] Additionally, HCV antibody testing cannot distinguish between resolved HCV (R-HCV) and viremic HCV (V-HCV), detect acute infections, or be reliably used in immunocompromised individuals.[17,18] Currently available HCV RNA testing assays, including point-of-care (POC) assays, are primarily lab based and costly, limiting access for populations disproportionately affected by HCV, such as AI/AN.[12,15,19] In this context, low-cost, rapid, sensitive, and specific POC HCV antigen (Ag) testing presents a promising alternative, offering a one-step solution for screening and diagnosis, particularly in resource-limited settings.[20–30] POC HCV Ag diagnostics serve as a reliable alternative for detecting active HCV infection and viral replication,[22–28] as HCV Ag can be detected in blood during the acute infection stage, well before antibodies become detectable.[30] However, there is currently no commercially available and US Food and Drug Administration (FDA)-approved POC HCV Ag testing device. Existing HCV Ag assays are primarily designed for laboratory use, making them expensive and lacking the necessary sensitivity and specificity, especially for samples with low viral loads (<1,000 IU/mL).[31–39] The limit of detection (LOD) for developed HCV Ag diagnostics ranges from 3,000 to 10,000 IU/mL, compared to the 12–15 IU/mL LOD of PCR-based assays.[39–41]

Bioluminescence imaging is a powerful technique in various scientific disciplines due to its non-invasive nature, low phototoxicity, and minimal background interference, making it ideal for longitudinal studies and targeting biological processes.[42,43] This method detects molecules in complex media, such as body fluids, with minimal sample handling and no need for external excitation light, overcoming limitations of gold-standard assays like ELISA[44] and addressing issues with fluorescence[45] and colorimetric[46] methods. Bioluminescence offers a reliable and robust biosensing option characterized by a high signal-to-background ratio, cost effectiveness, and user friendliness, making it suitable for bioanalytical applications, including POC diagnostics.[47,48] Advanced bioluminescence techniques can detect and quantify targets like small-molecule drugs and antibodies through bioluminescence resonance energy transfer (BRET), allowing precise quantification.[49,50] However, these systems require specific binder designs and extensive protein engineering for adequate BRET, while split-luciferase systems often face challenges such as reduced enzyme activity[51–54] and rapid signal decay, which complicates reproducibility.[55,56]

In this study, we report a highly sensitive and user-friendly automated POC HCV Ag assay utilizing enzyme cascade reaction-based bioluminescence imaging (Figure 1). This rapid magnetic bead (MgBead)-based immunoassay[57,58] enhances the bioluminescence signal by incorporating excess natural luciferase in the second enzyme cascade step,[59,60] generating high-yield luminescence without reducing enzyme activity. The cascade produces an intermediate substrate oxidized by luciferase, ensuring continuous bioluminescent signals[61,62] and reproducibility. Integrated into a portable system, this assay enables rapid sample-to-answer HCV analysis in under 30 min without external power, making it ideal for on-site diagnostics in underserved areas. The assay's sensitivity, simplicity, and portability position it as a promising alternative for expanding HCV testing access among high-risk populations, particularly AI/AN.

MATERIALS AND METHODS

Materials

All the reagents and solvents employed were commercially available and used as supplied without further purification. The Dynabeads Antibody Coupling Kit (14311D), streptavidin β-galactosidase (GAL) conjugate (S931), SiteClick Antibody Labeling Kits (S20033),

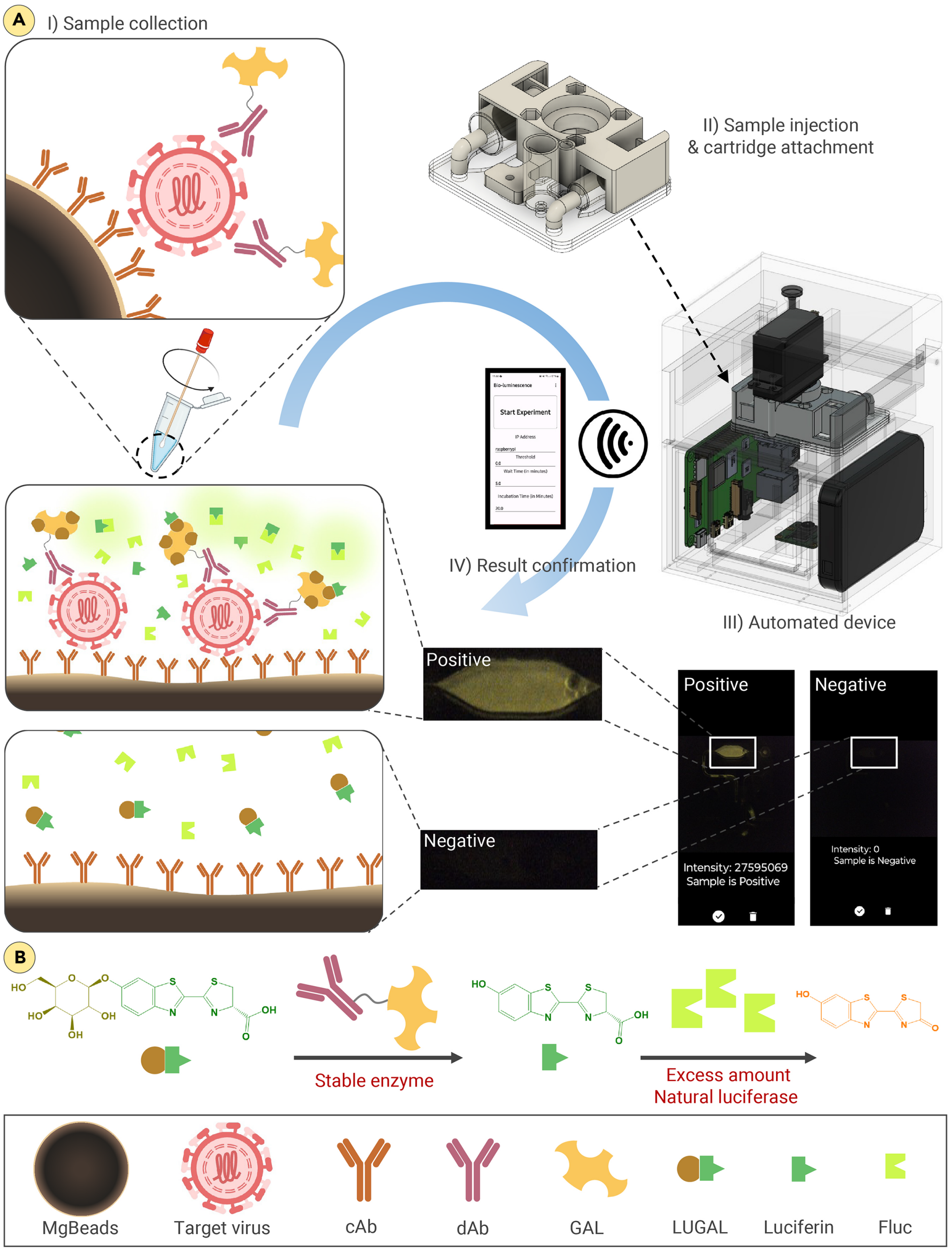

Figure 1. Schematic illustration of the enzyme cascade-based bioluminescence system for the detection of HCV Ag in fingerprick volume of plasma (A) Schematic overview of the automated enzyme cascade-based bioluminescence system: (I) collecting the sample and mixing it with assay materials, (II) transferring the mixture to a disposable microfluidic cartridge and inserting the cartridge to the detection module, (III) starting automated sample processing, signal generation, and data acquisition on a stand-alone optical reader with a custom-built smartphone application, and (IV) assay results reporting on the smartphone application. (B) Schematic illustration of the enzyme cascade system. The reaction is triggered by GAL, initiating the conversion of caged luciferin (LUGAL) to luciferin, followed by oxidation with Fluc, generating highly sensitive bioluminescence. LUGAL, D-luciferin-6-O-β-D-galactopyranoside; cAb-MgBeads, capture antibody-conjugated magnetic beads; dAb, detection antibody; GAL, β-galactosidase; Fluc, firefly luciferase.

MagJET Separation Rack for 12 × 1.5 mL tube (MR02), low protein binding microcentrifuge tubes (2 mL, 88380), 1-Step Slow 3,3′,5,5′-tetramethylbenzidine (TMB)-ELISA substrate solution (34024), and rabbit anti-human immunoglobulin (Ig)G (H+L) secondary antibody (horseradish peroxidase [HRP], A18903) were purchased from Thermo Fisher Scientific. The concentration of antibody was determined using the Pierce BCA Protein Assay Kit (Thermo Fisher Scientific, 23225). Adenosine 5′-triphosphate (ATP) disodium salt hydrate (A1852-1VL), Tris(2-carboxyethyl) phosphine hydrochloride, powder (≥98%, C4706-2G), ethylenediaminetetraacetic acid (EDS-100G), coenzyme A sodium salt hydrate (C4780-25MG), magnesium sulfate heptahydrate (230391-25G), luciferase from *Photinus pyralis* (firefly, SRE0045), and D-luciferin (L9504) were purchased from Sigma-Aldrich. D-luciferin-6-O-β-D-galactopyranoside (LUGAL; ab275053) was purchased from Abcam. Viral transport medium (VTM) was purchased from Innovative Research (IGVTM500ML). Tween 20 (BP337100) was purchased from Fisher Chemical. Tris-buffered saline with 0.1% Tween 20 detergent (TBST) 20× (pH 7.5, 40120065-1) was purchased from bioWORLD. The GAL antibody (HRP, GTX26646) was purchased from GeneTex. Flat-bottom 96W half-area UV-star plates were purchased from Thomas Scientific. Clear flat-bottom immuno nonsterile 96W plates were purchased from Thermo Fisher Scientific. A microplate reader (Infinite M nano+, Tecan) was used for TMB-ELISA. Poly(methyl methacrylate) (PMMA; 12 × 12 1.5 mm clear cast), O-rings, bolts (M3 × 0.5 mm), nuts (M3 × 0.5 mm), and ball bearing 6802 RS were purchased from McMaster-Carr. Double-sided adhesive (DSA; 3M optically clear adhesive 8146-2, 50 μm) was purchased from 3M. Syringes (1, 3, and 10 mm) were purchased from BD Syringe. Arduino Nano was purchased from AITEXM Robot. Raspberry Pi 4 was purchased from Raspberry Pi Foundation. The complementary metal oxide semiconductor (CMOS) sensor (Arducam IMX519 PDAF&CDAF autofocus camera module for Raspberry Pi) was purchased from Arducam. The servo motor (MG995 180°) was purchased from Towerpro. The "U shape type C male to female 40 Gbps connector" was purchased from AreMe. The "type A male to USB 3.1 type C male up opposite U shape back angled 90 degree charge adapter" was purchased from Xiwai. Jumper wires (male to female) were purchased from EDGELEC. A laser cutter (VLS3.60DT, Universal Laser Systems) was used for cutting PMMAs.

Preparation of the bioluminescence buffer

Following the protocol described in the reported article, a bioluminescence buffer was prepared as a working solution. Briefly, ATP (1.0 mM), BSA (7.6 mM), TCEP (1 mM), EDTA (0.1 mM), $MgSO_4 \cdot 7H_2O$ (2.0 mM), and coenzyme A (8.0 μM) were mixed in 9.8 mL of 1× Tris-HCl.

Preparation of working solution

LUGAL (5.0 μg/μL, 2.5 μL) and firefly luciferase (Fluc; 1.0 μg/μL, 50.0 μL) were mixed in 447.5 μL of bioluminescence buffer. After gentle mixing, 100 μL of the working solution was used for each assay.

Information of antibody pairs and recombinant Ag

Paired antibodies (MBS569238 for capture and MBS569240 for detection) for HCV core Ag (cAg) were purchased from MyBioSource, and recombinant HCV cAg (8903) was purchased from ViroStat.

Preparation of dAb-biotin

We used the SiteClick Antibody Labeling Kit (Thermo Fisher Scientific, S20033) to conjugate the detection antibody (dAb) with biotin (dAb-biotin) according to the manufacturer's guidelines. The main points of the SiteClick conjugation process are altering the antibody's carbohydrate domain, conjugating azide to the antibody, and then attaching it with the strained dibenzocyclooctyne (sDIBO)-modified biotin using copper-free click chemistry. This method results in the formation of a covalent bond between the biotin and the antibody, allowing us to obtain the dAb conjugated with biotin.

Investigation of dAb-biotin using TMB-ELISA

To confirm the function of dAb-biotin, we utilized TMB-ELISA. Initially, the surface of a 96-well plate was coated with 100 ng of HCV cAg and incubated at 4°C overnight.

Subsequently, the plate was washed four times with 200 μL of 1×TBST and then blocked with 200 μL of blocking solution (5% BSA in Milli Q) for 90 min at room temperature. After removing the blocking solution, the wells were incubated with or without 50 ng of dAb-biotin in 2.5% BSA in 1×TBST for 1 h at room temperature. Following four washes, the wells were incubated with 50 ng of streptavidin-HRP or 10 ng of anti-IgG-HRP in 2.5% BSA in 1×TBST for 1 h at room temperature. After five washes, 100 μL of TMB substrate was added, and the color change was observed for 20 min. Upon addition of 100 μL of stop solution (0.16 M sulfuric acid), the reaction was stopped, and the absorbance of each well was analyzed at 450 nm wavelengths.

Preparation of dAb-conjugated GAL

The dAb-biotin produced following the SiteClick Antibody Labeling Kit and streptavidin-conjugated GAL (ST-GAL; Thermo Fisher Scientific, S931) were mixed at a 1:4 mass ratio and diluted using Tris (pH 7.0, 0.05 M) to achieve a final antibody concentration of 25 ng/μL. This mixture was then incubated at 4°C for 2 days with gentle rotation. Following incubation, the conjugation of dAb-biotin with ST-GAL was confirmed by western blotting using anti-IgG-HRP and anti-GAL-HRP.

Investigation of dAb-conjugated GAL using TMB-ELISA

The surface of a 96-well plate was coated with 100 ng of HCV cAg and incubated at 4°C for overnight. Subsequently, the plate was washed four times with 200 μL of 1×TBST and then mixed with 200 μL of blocking solution (5% BSA in Milli Q) for 90 min at room temperature. After removing the blocking solution, the wells were incubated with 50 ng of dAb-conjugated GAL (dAb-GAL) in 2.5% BSA in 1×TBST or without it for 1 h at room temperature. After four washes, the wells were incubated with or without 50 ng of anti-GAL antibody in 2.5% BSA in 1×TBST for 1 h at room temperature. Following four washes, the wells were incubated with 100 ng of anti-human IgG-HRP or 10 ng of anti-mouse IgG-HRP in 2.5% BSA in 1×TBST for 1 h at room temperature. After five washes, 100 μL of TMB substrate was added, and the color change was observed for 20 min. After adding 100 μL of stop solution (0.16 M sulfuric acid), the absorbance of each well was analyzed using a microplate reader at 450 nm wavelengths.

Investigation of cAb-MgBead conjugation

To evaluate the function of capture antibody (cAb) conjugated on the surface of MgBeads, we mixed cAb-MgBeads (2 μL), MgBeads (2 μL) without cAb conjugation, cAb (0.4 μg) without MgBeads, or PBS (1 μL) with anti-IgG-HRP (0.01 μg in 100 μL of PBS). After 20 min incubation at room temperature, the mixtures were washed with 200 μL of 1×TBST, and the supernatant was removed. This washing step was repeated three more times. The pellet was resuspended with 100 μL of TMB, and absorbance was measured at 450 nm using a microplate reader after adding 100 μL of stop solution at the 20 min time point.

Preparation of VTM-passivated cAb-MgBeads

The cAb-MgBeads were prepared using the Dynabeads Antibody Coupling Kit (Invitrogen, 14211D), which utilizes an epoxy-amine reaction mechanism for antibody conjugation. According to the manufacturer's instructions, the reaction involved 5 mg of MgBeads and 100 μg of cAb. The final volume of the cAb-MgBead solution was 500 μL, which was divided into 5 aliquots of 100 μL each and stored at 4°C. For surface passivation of the cAb-MgBeads, 20 μL of the solution was incubated with 1.5 mL of VTM at 37°C for 30 min with gentle rotation. After incubation, the mixtures were fixed using a magnetic rack pull-down, and the supernatant was removed. The MgBeads were washed twice with 500 μL of 1×TBST. Following washing, the VTM-blocked cAb-MgBeads were stored in 20 μL of PBS.

Evaluation of the functionality of the individual components of the enzyme cascade-based bioluminescence system

We mixed 1 μL of cAb-MgBeads (0.2 μg of cAb in PBS), 1 μL of dAb-GAL (0.025 μg of dAb in 0.05 M Tris [pH 7.0]), and VTM with or without 100 ng of HCV cAg. After a 20 min incubation at room temperature, the supernatant was removed using a magnet to pull down the mixture. The resulting pellet was resuspended twice in 200 μL of 1×TBST. The washing step was repeated once more. The pellet was resuspended in 100 μL of working solution, and the bioluminescence signal was detected using a CMOS sensor.

Evaluation of clinical LOD

Clinical samples of HCV were serially diluted in plasma to concentrations of 1.5×10^7, 1×10^6, 1×10^5, 1×10^4, 5×10^3, 1×10^3, and 5×10^2 copies/mL. Prior to testing, a lysis step was performed for the HCV clinical samples by incubating them at room temperature with 0.1% Tween 20 for 1 min. The samples were then mixed with assay materials and processed following the protocol outlined in the "System integration: A fully automated enzyme cascade-based bioluminescence system" and Figure S5. The resulting bioluminescence intensities under each condition were used as references to determine the LOD of the system. We conducted n = 5 technical replicates, and the LOD of the automated enzyme cascade-based bioluminescence system was calculated as the mean of the blank plus three times the standard deviation (SD) obtained for the blank.

Development of smartphone applications

In our detection module, the Raspberry Pi was the central processing unit and host controller and served as the core computational unit responsible for executing the image processing algorithms and coordinating the data acquisition process. To facilitate the interaction between our image processing system and the user, we developed an Android application using Android Studio (v.2022.2.1). This Android app enabled seamless communication with the Raspberry Pi and allowed us to remotely trigger the sample insertion and image capture process while maintaining incubation time and waiting time. The communication between the Android app and the Raspberry Pi was established through a Flask server hosted on the Raspberry Pi. This server acted as the intermediary, facilitating the exchange of commands and data between the two devices. In addition to its role as a computational unit, the Raspberry Pi also controlled the physical components of our setup. To send samples into the imaging system at the designated times, we integrated servo motors controlled by an Arduino board. The Raspberry Pi communicated with the Arduino to precisely control the movement of these servo motors, ensuring the accurate delivery of samples into the microfluidic channel setup. This level of automation and control enhanced the consistency and reliability of our image capture process. We utilized a Pi camera module to capture images from Raspberry Pi. For image processing, our primary goal was to assess where the sample was positive or negative using the bioluminescence property of the sample. To achieve this, we utilized OpenCV v.4.8.0, a widely used computer vision library that provides essential tools for image processing. The approach involved several steps. First, we cropped the images to isolate the region of interest. This cropping ensured that our analysis focused exclusively on the relevant areas. Next, we converted the cropped images to the HSV (hue, saturation, value) color space. To detect the target color pattern—in this case, yellow—we defined the lower and upper bounds within the HSV color space that corresponded to the desired color range. We then created a mask to extract the pixels falling within these boundaries. To refine our results and remove unwanted noise, we applied morphological operations to the mask, namely closing and opening operations. Subsequently, we used the mask to segment the relevant region within the HSV image. By applying this mask, we effectively isolated the yellow color pattern within the region of interest. This segmentation step was crucial for our analysis. Finally, we calculated the brightness of the segmented yellow region by summing the values in the value channel of the HSV image. This value served as our quantitative measure of the bioluminescence property of the sample.

Specificity test

The specificity of the enzyme cascade-based bioluminescence assay was evaluated using AI/AN HCV-spiked plasma samples and non-target samples, including HIV and HBV, each at virus concentrations of approximately 1×10^6 copies/mL. The assay was performed following the automated assay protocol described in Figure 3, and the intensity of the bioluminescence was quantified using a smartphone application.

Biosafety and human participant statement

The research work reported was approved and performed in adherence to guidelines and procedures approved by the institutional biosafety committee of Mass General Brigham (the parent organization of Massachusetts General Hospital and Brigham and Women's Hospital) and under appropriate institutional review boards (IRB nos. 2015P000454, 2019P002209, 2019P001996, 2019P001489, and 2023P000538). This study was approved by the Cherokee Nation IRB. Patient enrollment was voluntary, and informed consent was required for participation. Except for the member of Cherokee Nation Health Services, we do not have access to identifiable information. All the experiments with HCV were performed in BSL2+. AI/AN HCV-infected patient plasma samples were provided by the Cherokee Nation. HBV-infected patient serum/plasma samples were purchased from Discovery Life Sciences. The viral loads of HBV-infected and HCV-infected patient serum samples were measured using a standard RT-PCR system and reported by Mass General Hospital and the vendor (Discovery Life Sciences). HIV stock-cultured samples were received from our colleagues at the division of infectious diseases at Brigham and Women's Hospital. The spiked samples that were used in system testing throughout the study were prepared by serial dilutions of stock virus samples with known viral loads measured by standard PCR approaches. In these cases, the control samples were virus-free samples that were

processed for the detection assay with the same protocol as the viral samples, except for the addition of viral-stock dilutions.

Statistical analysis

Statistical analyses were performed using Origin software v.8.5 (OriginLab, Northampton, MA, USA). All viral load values were converted into log10 copies/mL. The mean and SDs were calculated for each data point from at least a total of 2–3 independent experiments unless indicated in the text. The level of significance was set at $p > 0.05$. Receiver operating characteristic (ROC) and scattering plot analyses were used to define the performance of the enzyme cascade-based bioluminescence system to the standard analytical technique. The agreement between categorical variables was evaluated using Cohen's κ coefficient.

Large language models

The authors exclusively authored the entire manuscript. Large language models (LLMs), such as ChatGPT, were solely utilized for addressing typographical errors and making text edits.

RESULTS

Materials characterization

To adapt the automated enzyme cascade-based bioluminescence system for POC testing, we synthesized the assay materials, including the site-specific biotinylation of the dAb (dAb-biotin), using the Invitrogen SiteClick biotin antibody labeling kit (S20033; Thermo Fisher Scientific) following the manufacturer's instructions. The conjugation was confirmed through ELISA, which demonstrated a distinct color change of TMB specifically in the presence of dAb-biotin treated with streptavidin-HRP. In contrast, no color change was observed for the unconjugated antibody. This clear distinction indicates that the biotin conjugation on the antibody does not impair its functionality toward target proteins, and the attached biotin molecules remain accessible for subsequent streptavidin binding (Figure S1). Following site-specific modification, we assessed the recovery yield of the dAbs using the BCA (bicinchoninic acid) assay, which revealed conjugation yields of 92% for the HCV antibody.

The synthesis of the dAb-GAL conjugate was achieved through a straightforward procedure by mixing dAb-biotin with ST-GAL (Figure 2A).[63,64] The successful conjugation of dAb-GAL was confirmed with western blotting, where protein bands detected with anti-IgG-HRP precisely matched those detected with anti-GAL-HRP, validating the synthesis of dAb-GAL (Figure 2B). The functionality of the dAb-GAL conjugate was further assessed using a TMB-ELISA. In this assay, a distinct color change was observed when dAb-GAL was treated with the HCV cAg. In contrast, no color change was noted in the absence of ST-GAL, dAb-GAL, or the cAg. These results confirm that the dAb-GAL conjugate retains its functional activity (Figures 2C and 2D).

To prepare the cAb-MgBeads, we employed the Dynabeads Antibody Coupling Kit according to the supplier's instructions (Figure 2E). The concentration of cAb covalently coupled to the MgBeads' surface was quantified using a BCA assay, yielding 14.9 μg/mg. To block the surface of the conjugated MgBeads and prevent non-specific binding, the cAb-MgBeads were further incubated with VTM (materials and methods). The successful conjugation of cAb on the MgBeads' surface was confirmed by the color change of TMB upon the addition of anti-IgG-HRP. Specifically, a yellow color change in TMB was observed in the presence of cAb-MgBeads, whereas no change was detected when MgBeads without cAb conjugation were used (Figure 2F). This confirms the successful conjugation of cAb on the MgBeads' surface.

Following the preparation and optimization of assay materials, we evaluated the efficacy of the automated enzyme cascade-based bioluminescence system in capturing a target protein and initiating bioluminescence using an immunoassay, using recombinant HCV cAg as a clinical model. Strong bioluminescence was observed in the presence of all components, while no signal was detected in the absence of the target Ag (Figure 2G). These results affirm the efficient capability of the selected dAbs to capture the target molecule and indicate that ST-GAL, conjugated to the dAb, initiates a cascade reaction leading to bioluminescence production.

System integration: A fully automated enzyme cascade-based bioluminescence system

A fully automated enzyme cascade-based bioluminescence system was developed, incorporating a microfluidic cartridge with pre-loaded reagents and a portable, cost-effective reader designed for simple sample handling. By pressing a single button on a custom smartphone application, all essential procedures, including washing sequences, signal generation, data capture, and result reporting, were seamlessly initiated and managed (Figure 3A).[57]

The hardware part of the assay comprises a disposable microfluidic cartridge and a reusable standalone detection module, both fabricated using a 3D printer and a laser cutter, as illustrated in Figures 3B and 3C. The disposable microfluidic cartridge was fabricated by laser cutting three identical 1.5-mm-thick PMMA substrates (Figures 3B and 3D). These substrates were then assembled using DSA. Additionally, the cartridge included a 3D-printed chip cover made from polylactic acid (PLA), incorporating a rotation valve, a magnet for the reaction chamber, and syringes connected to the inlets. The cartridges also included two pre-loaded chambers: one containing 400 μL of washing buffer (1×TBST) and the other containing 150 μL of the working solution. Additionally, the cartridge has a separate reaction chamber where the assay is conducted. The cartridge features a magnet attached to one side of the reaction chamber. The cartridge was designed with three distinct inlets, allowing for the injection of the sample, washing buffer, and working solution. These inlets are connected to the reaction chamber through a network of channels. The flow between these channels and the reaction chamber is controlled by a small connecting channel within the rotation valve, allowing the sequential entry of each solution into the reaction chamber at specific stages of the assay.

The standalone optical reader, measuring 122 × 112 × 158 mm, was specifically designed to fully encompass the microfluidic cartridge (Figures 3C and 3E). It effectively blocks external light, creating a dark environment for the precise monitoring of bioluminescence. The standalone optical module comprises a body and a lid that are attached to each other using two M3 bolts on each side. Within the module, various components are housed, including the cartridge, Raspberry Pi 4, Arduino Nano, servo motor, battery pack, and a CMOS image sensor. The CMOS sensor is centrally positioned at the bottom of the module, precisely 56.5 mm below the cartridge, to capture a focused image of the bioluminescence signal within the assay chamber. To automate the assay process, a servo motor is mounted on the lid with a rotation valve lever connected to it. The Raspberry Pi sends signals to the Arduino Nano, which activates the servo motor. The lever attached to the motor then pushes the syringe into the chamber containing the buffer solution. Additionally, the Raspberry Pi controls the CMOS sensor to capture images with the desired parameters for further analysis.

A multistep approach was implemented to optimize the performance of the device and assay. Initially, the optimization focused on the allowed air volume in multiple components of the device in order to deliver the correct volume of various reagents reliably (Figure S2). Proper device function is dependent on the correct distribution of reagents with air pressure control. Based on this analysis, the optimal air volumes were determined to be 0.3 mL for sample transfer, 1.2 mL for wash buffer removal, and 0.2 mL for working solution injection (Figure S2). Too small of a volume for the washing buffer ejection (<1.2 mL) diminished the final assay signal due to interference from the remaining wash buffer (Figure S3). Volumes as low as 10 μL of residual washing buffer (1×TBST) can result in a weaker bioluminescence signal (Figure S3). Therefore, it is crucial to thoroughly remove the washing buffer after sample washing to ensure accurate and reliable results.

To further reduce the chance of signal interference, a blocking agent, VTM, was applied to the cartridge chamber and channels. cAb-MgBeads and dAb-GAL were incubated in the assay chamber under assay conditions without viral biotargets. After washing, the working solution was added to assess the occurrence of bioluminescence. Cartridges treated with the VTM showed no bioluminescent signals, whereas untreated cartridges exhibited detectable bioluminescent signals, demonstrating the value of VTM blocking to prevent false positives (Figure S4).

The functionality of the automated system was initially assessed using three distinct food-colored dyes: red to represent the sample with cAb-MgBeads and dAb-GAL, blue for the washing buffer (400 μL of 1×TBST), and green for the working solution. The process begins by mixing the collected sample with assay materials (red) and transferring the mixture to the assay chamber within the microfluidic cartridge. By opening the smartphone application and pressing the start button, the sequence is initiated, starting a 20-min incubation period within the assay chamber. After incubation, a rotating valve automatically pivots

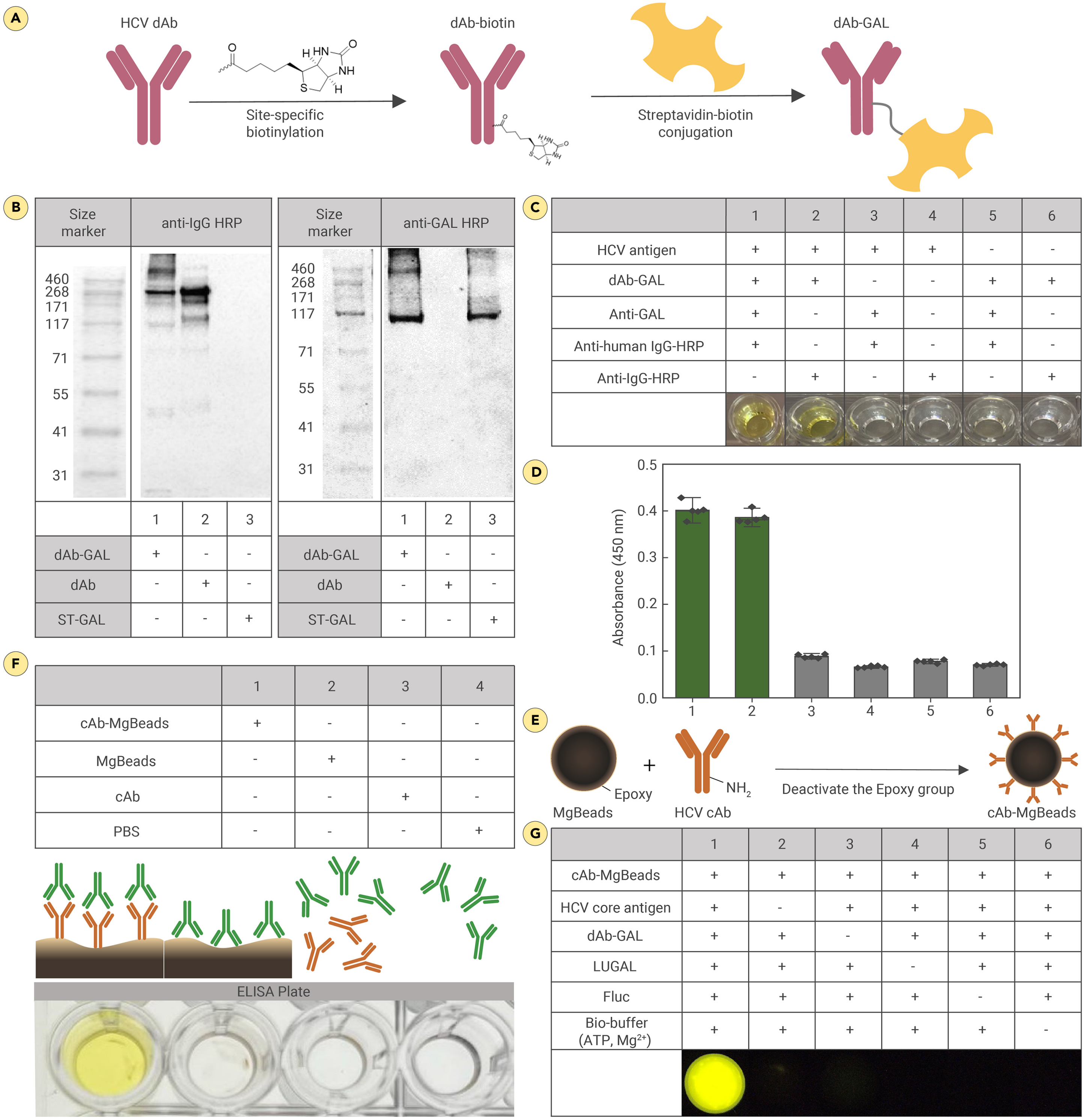

Figure 2. Assay materials characterization (A) Schematic presentation of the protocol used in the preparation of dAb-GAL. Biotin molecules were conjugated to the Fc region of dAb through a site-specific conjugation method. Following the biotin conjugation, ST-GAL was bound to the biotin through streptavidin-biotin interaction. (B) Western blot of the prepared dAb-GAL. dAb-GAL was characterized by western blot with anti-IgG-HRP and anti-GAL-HRP. Lane 1: dAb-GAL; lane 2: dAb; lane 3: ST-GAL. (C and D) Experimental condition (C) and results of ELISA (D) for dAb-GAL. Data are mean ± SD with n = 5 technical replicates. (E) Schematic presentation of the protocol used in the preparation of cAb-MgBeads. (F) Magnetic pull-down assay for cAb-MgBeads. (G) Evaluating functionality of the individual components of the system. Anti-GAL, anti-β-galactosidase; ATP, adenosine 5′-triphosphate.

clockwise to align with the chamber containing the blue dye (washing buffer). The valve then propels a syringe loaded with washing buffer to wash the captured mixture, expelling excess washing solution and air into a drain chamber. Following the washing step, the rotating valve shifts counterclockwise to access the chamber housing the green dye (working solution). It injects a syringe filled with the working solution into the chamber, triggering bioluminescence, which is captured by the CMOS sensor for result analysis. The captured image is analyzed within the smartphone application to determine the sample's positive or negative status. The entire sample-to-answer assay process is completed in approximately 23 min, including the virus capture incubation time. Video S1 provides a comprehensive demonstration of the automated sample handling process performed on the cartridge.

The automated assay protocol for the enzyme cascade-based bioluminescence system is as follows. Step 1: the test sample is introduced into an

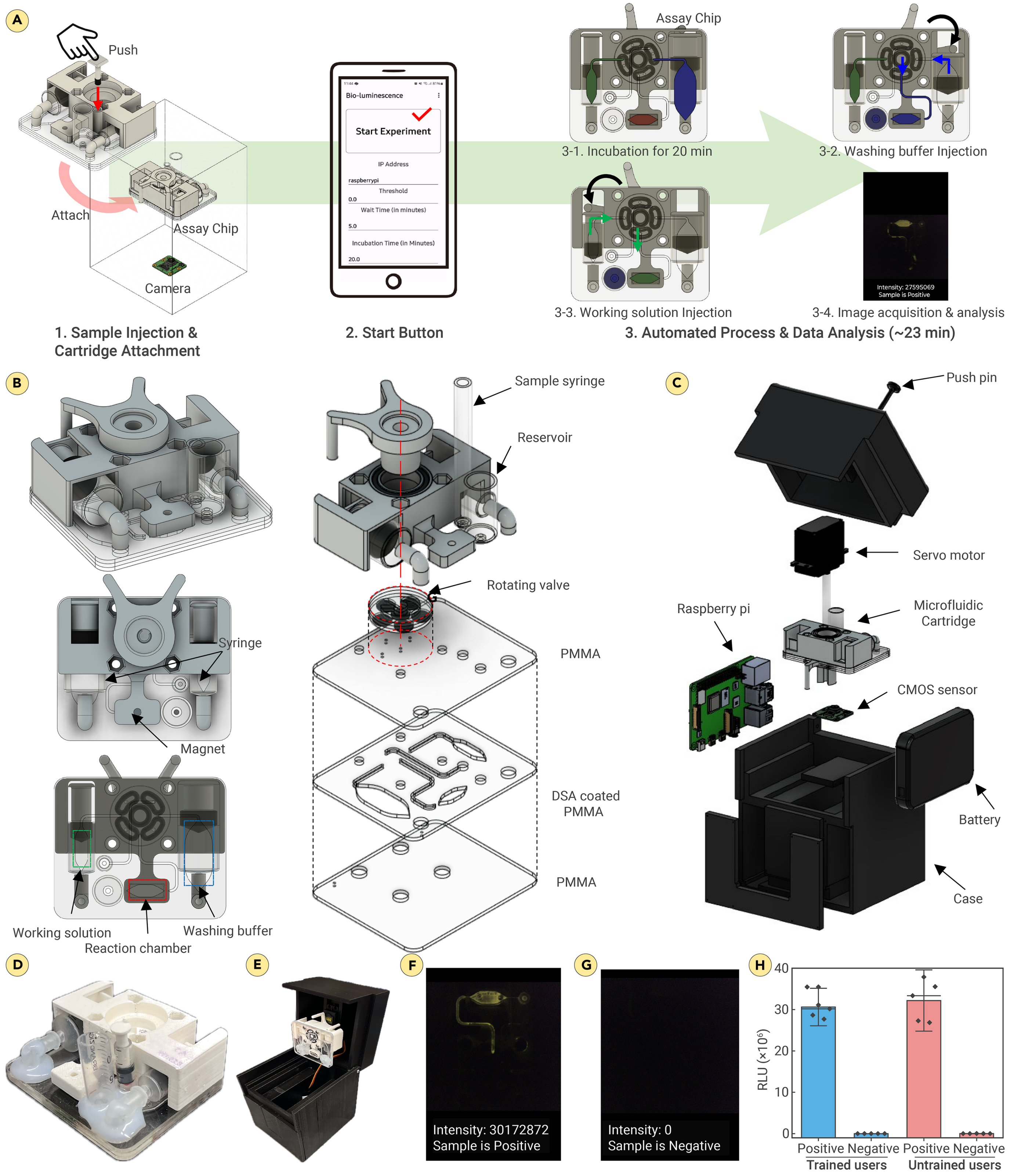

Figure 3. Fully automated HCV Ag assay (A) Schematic illustration of the automated assay process. (1) Sample is injected into the cartridge through an injection hole. (2) The sample processing and signal generation are initiated by pressing a "start" button in a custom-built smartphone application. (3) Target HCV cAg capture, washing steps, signal generation, and data acquisition through a built-in CMOS sensor are done automatically. (B) The design and exploded view of the microfluidic cartridge. (C) The exploded view of the detection module for automated assay process. (D and E) The actual images of the microfluidic cartridge (D) and the detection module (E). (F and G) Validation of the assay with (F) or without (G) spiked HCV cAg. (H) Usability test. A total of 10 users were involved, comprised of five trained individuals and five untrained users with diverse backgrounds. The t test analysis revealed no significant difference between the results obtained by the trained and untrained participants ($p = 0.56$ for positive and $p = 0.35$ for negative). Bars indicate mean ± SD for $n = 5$ independent users per group.

Eppendorf tube containing cAb-MgBeads (0.4 μg of cAb in PBS) and dAb-GAL (0.05 μg of dAb in 20 mM Tris-HCl) and is swirled for 30 s. The mixture is transferred to the sample injection syringe. Step 2: a disposable pipette is used to transfer the mixture of the sample and assay materials into a disposable microfluidic cartridge. An injection bar is used to push the solution into the microfluidic cartridge. Step 3: the disposable cartridge is inserted into the reusable

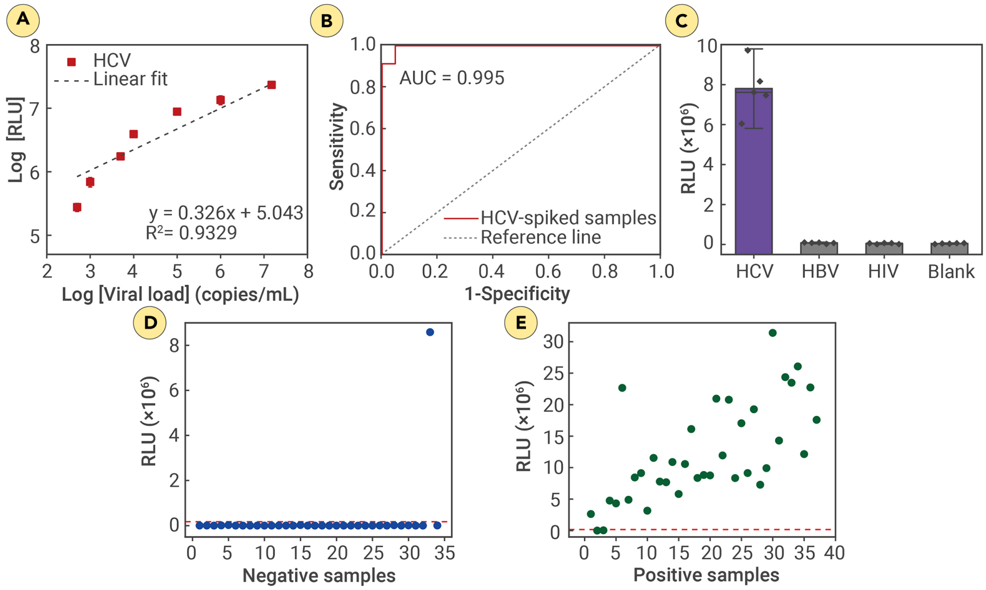

Figure 4. Assay validation with AI/AN HCV-spiked plasma samples and HCV Cherokee Nation patient samples (A) Dose-response curve of the bioluminescence intensity (RLU) of the automated assay against different concentrations of serially diluted HCV-spiked samples. AI/AN patient HCV plasma samples were serially diluted with plasma to generate samples with virus concentrations of 1.5×10^7, 1×10^6, 1×10^5, 1×10^4, 5×10^3, 1×10^3, and 5×10^2 copies/mL. A lysis step was employed prior to testing (incubation at room temperature with 0.1% Tween 20 for 1 min). The samples were mixed with assay materials and processed according to the protocol described in the automated assay protocol of the enzyme cascade-based bioluminescence system. Data are mean ± SD with $n = 5$ technical replicates. (B) ROC analysis of the enzyme cascade-based bioluminescence system using biological replicate of HCV-spiked plasma samples ($n = 92$). The experimental LOD of the automated assay was determined by obtaining the cutoff value from the ROC curve and then applying that value to the trend lines of (A). (C) Specificity test using target and non-target samples including HIV and HBV. Bars represent mean ± SD with $n = 5$ technical replicates. Blank is pure sample buffer. (D and E) Vertical scatterplots using HCV-positive (D) and -negative (E) patient samples collected from AI/AN in the Cherokee Nation. Red dashed line indicates threshold determined by the ROC curve of HCV-spiked samples.

standalone detection module. Step 4: the "start" button is pressed in the smartphone application to initiate the automated sample handling (Figure S5).

The performance of the fully automated assay was first evaluated using samples containing recombinant HCV cAg. Evident and vibrant bioluminescent signals were observed in the presence of the target protein, accompanied by a positive indication on the smartphone application, as depicted in Figure 3F. Conversely, in the absence of the target virus, no bioluminescent signal was detected, and a negative result was displayed on the smartphone application (Figure 3G).

The microfluidic cartridge was designed for single use, while the detection module can be employed repeatedly for multiple tests. The material costs for manufacturing the microfluidic cartridge amount to approximately $2.30, while the cost for the detection module is approximately $86.84 (Table S1). These findings validated the successful integration of all facets of the reported automated enzyme cascade-based bioluminescence technology, encompassing sample handling, signal amplification, and readout, within a portable and cost-efficient cartridge.

To assess the frequency of user errors during sample processing using the fully automated system, we conducted experiments involving a diverse group of individuals. These tests utilized samples containing the recombinant HCV cAg as the test material. The participant pool comprised a total of 10 individuals: five with specialized training and expertise related to the developed enzyme cascade-based system and five untrained individuals with diverse backgrounds, including non-PhD graduates and undergraduate students (Figure 3H). The untrained participants were provided with a user instruction sheet as their only reference to conduct the testing procedure (Figure S5; Video S2). The t test analysis revealed no significant difference between the results obtained by the trained and untrained participants ($p = 0.56$ for positive and $p = 0.35$ for negative), underscoring the user-friendly nature of the automated enzyme cascade-based system. This outcome emphasizes the ease of operation and the robustness of the automated assay, making it accessible and effective for a broad spectrum of users.

Assay validation with HCV-spiked plasma samples

Initially, we assessed the LOD of the automated enzyme cascade-based bioluminescence system by conducting experiments with serially diluted AI/AN HCV samples at virus concentrations of 1.5×10^7, 1×10^6, 1×10^5, 1×10^4, 5×10^3, 1×10^3, and 5×10^2 copies/mL (Figure 4A). The R-squared value was estimated at 0.9329, indicating a robust linear correlation between the bioluminescence intensity from the enzyme cascade-based system and the viral load measured by PCR. Figure 4A illustrates the system's performance across the clinically relevant concentration range for patient samples. While the overall trend is not strictly linear, the system demonstrates a strong linear response within the dynamic ranges of 500–10^4 and 10^5–10^7 copies/mL (Figure S6), each exhibiting an R-squared value greater than 0.99. These results highlight the system's ability to maintain high accuracy and linearity across the full spectrum of potential sample concentrations, ensuring reliable performance for clinical applications.

To determine the experimental LOD, we conducted ROC curve analysis using 92 serially diluted samples spiked with AI/AN HCV, covering virus concentrations ranging from 0 to 1.5×10^7 copies/mL (Figure 4B; Table S2). The ROC analysis revealed that a threshold HCV viral concentration of 768 copies/mL provided an optimal sensitivity of 97% (confidence interval [CI], 88.43%–100.00%) and specificity of 95% (CI, 83.30%–98.21%) for the enzyme cascade-based system in identifying viral infection ($n = 92$). The overall accuracy was 96% (CI, 89.24%–98.80%), with an area under the curve (AUC) of 0.995 and a binomial exact CI ranging from 0.9871 to 1.0000. The threshold value represents the experimental LOD of the enzyme cascade-based bioluminescence system for the detection of HCV viral pathogens.

The specificity of the assay was evaluated using AI/AN HCV-spiked plasma samples and non-target samples, including HIV and HBV, each at virus concentrations of approximately 1×10^6 copies/mL (Figure 4C). The bioluminescence of the non-target viral samples produced an intensity equal to or less than 7.5×10^4 relative light units (RLUs), comparable to the blank without any target virus present. In contrast, the HCV-spiked sample with the viral load of 1×10^6 copies/mL exhibited bioluminescence intensity exceeding 7 million RLUs. These findings demonstrate the specificity of the enzyme cascade-based system toward the target HCV virus.

Assay validation with AI/AN HCV patient samples

The developed automated POC HCV Ag assay was evaluated using 71 AI/AN HCV patient plasma samples, including 37 positive (viral load ≥ 768 copies/mL) and 34 negative (viral load < 768 copies/mL) patient samples (Table S3). The vertical scatterplots display the correlation between the automated enzyme cascade reaction results and PCR qualitative outcomes for patient samples and highlight specific discrepancies, which included 1 false positive and 2 false negatives (Figures 4D and 4E). The specificity, sensitivity, and accuracy of the POC HCV Ag assay in qualitatively classifying viral-infected samples with a clinically relevant viral load threshold of 768 copies/mL were 94%, 97%, and 96%, respectively.

The Cohen's κ coefficient was employed to evaluate the agreement and reliability between the two diagnostic results: PCR and the enzyme cascade-based bioluminescence system. The coefficient is a probability value, and the closer it is to 1, the higher the agreement between two different datasets. The coefficient turned out to be $\kappa = 0.875$, which indicates that the concordance between the two systems is almost perfect (Figure S7).

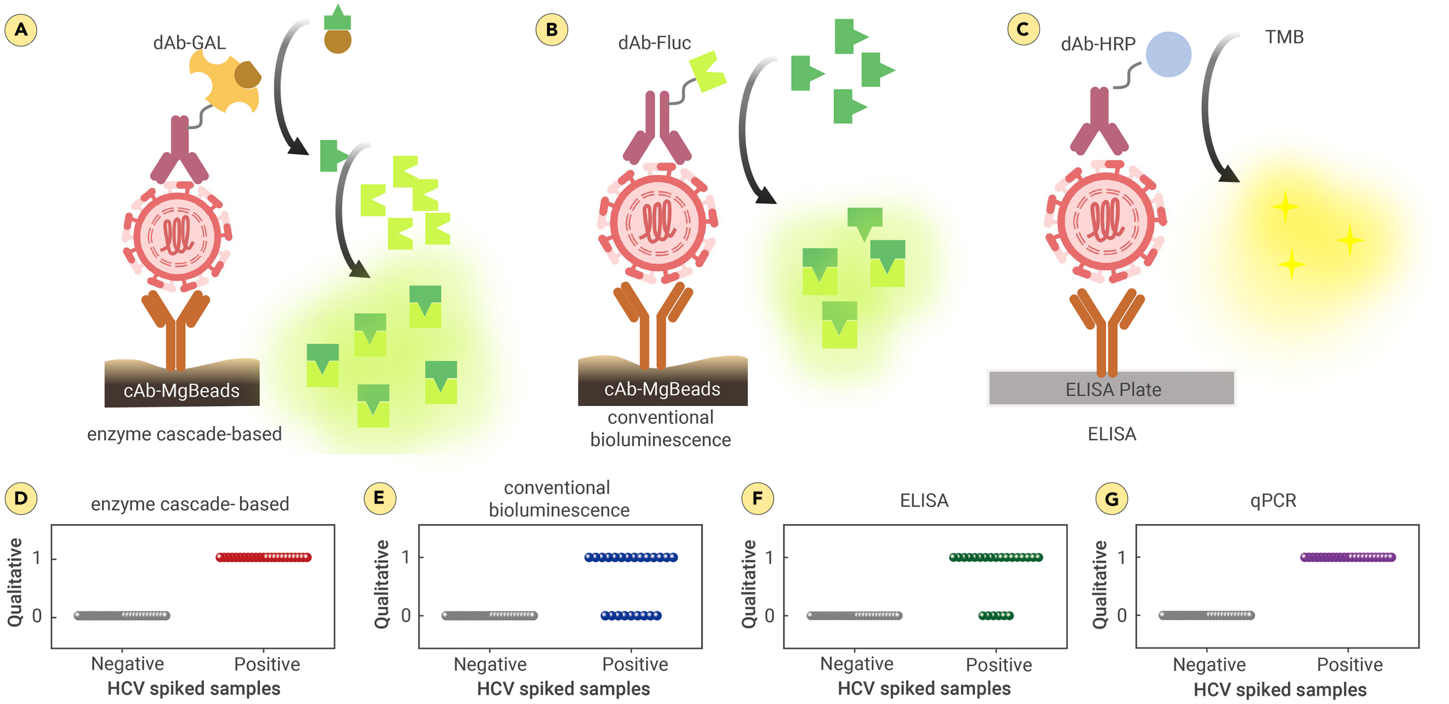

Figure 5. The performance of the developed POC HCV Ag assay compared to conventional bioluminescence-based approach and ELISA (A–C) Schematic illustration of enzyme cascade-based system (A), conventional bioluminescence system (B), and ELISA (C). Vertical scatterplots for qualitative classification of AI/AN HCV-spiked plasma samples with a viral load threshold of 768 copies/mL. (D–G) Vertical scatterplots of system evaluations using HCV-spiked samples with enzyme cascade-based POC HCV Ag assay (D), conventional bioluminescence system (E), ELISA (F), and qPCR (G). 0 indicates negative by the assay (viral load < 768 copies/mL) and 1 indicates positive by the assay (viral load ≥ 768 copies/mL).

Head-to-head comparison of the automated POC HCV Ag assay with conventional bioluminescence system and ELISA

To emphasize the advantages of the reported automated enzyme cascade-based bioluminescence system over other assays, we conducted a performance evaluation comparing it with a conventional bioluminescence system and ELISA. We evaluated the performance of each approach by calculating the clinical sensitivities and specificities in qualitatively classifying 50 AI/AN HCV-spiked plasma samples (23 positive and 27 negative samples) with a clinically relevant viral load threshold of 768 copies/mL[58,59] (Figures 5D–5F; Tables S4–S7). The clinical sensitivities and specificities were confirmed as follows: 61% and 100% for the conventional bioluminescence system and 74% and 100% for ELISA, respectively. The sensitivity and specificity of the enzyme cascade-based bioluminescence were both 100%. Accuracy was also evaluated, yielding 100% for the enzyme cascade-based bioluminescence, 78% for the conventional bioluminescence system, and 84% for the ELISA (Table S8). These results demonstrated that the reporting system outperformed the conventional bioluminescence system and ELISA when using a low viral load threshold. The enzyme cascade-based bioluminescence system was evaluated against qualitative qPCR, the gold standard, and showed equivalent sensitivity, specificity, and accuracy in detecting HCV samples at a viral load threshold of 768 copies/mL (Figure 5G).

DISCUSSION

The 2018 European Association for the Study of the Liver (EASL) guidelines and WHO Global Hepatitis Report recognize HCV Ag testing as an alternative to HCV RNA testing, particularly in resource-limited settings.[60,61] While the ARCHITECT HCV Ag assay is provisionally accepted for detecting acute HCV infection, the WHO notes its limitations in identifying individuals with low viral loads (<1,000 IU/mL).[20,62] The Abbott Architect HCV cAg assay showed a sensitivity of 12.5% for viral loads <12 IU/mL, 13.0% for viral loads = 12–999 IU/mL, and 81.9% for viral loads = 1,000–9,999 IU/mL.[37,38] In a recent study involving 744 clinical cases, the sensitivity of the HCV Ag test was reported as 82.1%.[20] Current serological assays for HCV detection fail to provide accurate, sensitive, specific, low-cost, and user-friendly POC detection of HCV Ag using fingerprick whole-blood samples.[65] For instance, the lateral flow assay developed by Wang et al. demonstrated only 10.71% sensitivity compared to RNA-based assays for samples with viral loads between 10^3 and 10^4 copies/mL.[66] There is an urgent need for cost-effective, scalable diagnostics for on-site, rapid HCV Ag testing, particularly for high-risk populations like AI/AN.[67] Implementing rapid POC HCV tests could enhance HCV management by facilitating immediate treatment decisions and reducing patient loss to follow-up.[9,68] This clinical need for POC HCV Ag diagnostics was discussed by national experts during a webinar hosted by the Association of Public Health Laboratories (APHL) in collaboration with the CDC's Division of Viral Hepatitis in October 2021.[69]

We have demonstrated that the enzyme cascade-based bioluminescence system, integrated into a fully automated assay device, can accurately detect HCV infection within 30 min, including sample incubation, washing steps, signal development, and analysis. The assay employs a self-contained, disposable cartridge design, with all required reagents pre-loaded into isolated reservoirs within the cartridge. This closed-system approach ensures that all assay processes occur entirely within the cartridge, preventing any external exposure or contamination. The standalone optical reader, which interacts with the cartridge, remains free from contact with the sample or reagents, preserving its integrity and eliminating concerns about contamination. This design effectively prevents cross-contamination between samples, as residual material from prior tests cannot affect subsequent assays, ensuring consistent accuracy, reliability, and reproducibility in results. In a study involving 71 AI/AN HCV patient samples, the system achieved 97% sensitivity, 94% specificity, and 96% accuracy, highlighting its exceptional performance in detecting HCV infections within AI/AN populations. Usability testing revealed no significant differences between user groups (p = 0.56 for positive results and p = 0.35 for negative results), confirming the system's intuitiveness, minimal user errors, and overall user friendliness. These features ensure that even untrained users can effectively operate the system, supporting its potential for widespread deployment. By providing rapid and accurate HCV diagnosis, this system can address health disparities and enable timely and effective treatment in underserved communities, such as AI/AN populations. The ability to diagnose HCV quickly and reliably is essential for improving patient outcomes and reducing virus transmission.

The cascade reaction system overcomes the limitations of traditional diagnostic methods, such as colorimetric assays, ELISA, and fluorescence-based assays, by offering high sensitivity, accuracy, and ease of use. Traditional systems often suffer from low sensitivity, complex assay processes, and the need

for expensive equipment and expertise. In contrast, this automated solution completes the assay within 30 min without requiring an external power supply, making it particularly valuable in resource-limited settings. The system's simplicity and robustness facilitate deployment in various clinical and field environments, enabling healthcare providers in remote or resource-poor areas to deliver high-quality diagnostic services. This versatility enhances healthcare access and equity, especially for underserved populations. Compared to other POC diagnostic tools, including POC HCV RNA testing systems, our platform offers a more rapid and user-friendly approach. For example, POC RNA-based systems often require more sophisticated equipment and involve longer assay times, limiting their applicability in decentralized settings. By streamlining the diagnostic process, this system reduces the operational complexity and time to diagnosis, addressing critical barriers to HCV screening and care. Furthermore, while other assays may focus solely on detecting viral RNA or Ags, the cascade reaction system provides a balanced combination of speed, sensitivity, and adaptability, ensuring accurate results without compromising ease of use. The ability to quickly diagnose HCV in resource-limited settings not only aids in immediate patient management but also plays a vital role in linkage to care. By enabling earlier identification of HCV-infected individuals, this system supports timely treatment initiation, reducing disease progression and transmission risks. The platform's integration with existing healthcare workflows can also enhance patient retention throughout the care continuum, bridging gaps that frequently occur with centralized laboratory testing. Moreover, the adaptability of this platform extends beyond HCV diagnostics, as it can be repurposed for other infectious diseases, such as HIV, HBV, and respiratory viruses, by modifying the antibody pair for the target pathogen. This potential for broader applications underscores its promise as a transformative diagnostic tool for global healthcare challenges. Exploring synergies with other POC diagnostics, including multiplexed assays, could further enhance its utility, providing comprehensive diagnostic solutions that address multiple healthcare needs in a single, streamlined workflow.

RESOURCE AVAILABILITY

Materials availability

All unique/stable reagents generated in this study are available from the lead contact with a completed materials transfer agreement.

Data and code availability

Data are available from the corresponding author upon reasonable request.

FUNDING AND ACKNOWLEDGMENTS

This work was partially supported by the National Institutes of Health under award numbers R01AI187513, R01EB033866, R01AI138800, R01AI138800-05S1, U54HL119145, R01HD115677, R33AI140489, and R61AI140489, all to H.S.

AUTHOR CONTRIBUTIONS

S.K. and H.S. designed the project. H.S. supervised the overall study. S.K., A.C., and J.C. performed the experiments. J.M.H. and J.L. developed the automated devices. S.K., A.C., and J.C. performed the data analysis. M.K.K., P.T., and H.K. developed the smartphone application. G.P.F. and J.G. performed the patient sample preparation at Massachusetts General Hospital. R.T.C. and J.M. provided HCV samples and HCV virology-related clinical advice. S.K., J.C., J.H.M., and H.S. wrote the manuscript. All co-authors edited the manuscript.

DECLARATION OF INTERESTS

H.S. and S.K. have filed a patent on the reported technology through Brigham and Women's Hospital.

SUPPLEMENTAL INFORMATION

It can be found online at https://doi.org/10.1016/j.xinn.2025.100952.

LEAD CONTACT WEBSITE

https://shafieelab.bwh.harvard.edu/

REFERENCES

1. WHO (2021). Global progress report on HIV, viral hepatitis and sexually transmitted infections, Accountability for the global health sector strategies 2016–2021: actions for impact, Available at: https://www.who.int/publications/i/item/9789240027077.
2. Mera, J., Vellozzi, C., Hariri, S. et al. (2016). Identification and Clinical Management of Persons with Chronic Hepatitis C Virus Infection - Cherokee Nation, 2012-2015. *MMWR Morb. Mortal. Wkly. Rep.* **65**:461–466. DOI:https://doi.org/10.15585/mmwr.mm6518a2.
3. CDC (2019). Hepatitis tables and figures - US 2017 surveillance data for viral hepatitis, Availabe at: https://www.cdc.gov/hepatitis/statistics/2017surveillance/index.htm.
4. Gutierrez, J.A., Lawitz, E.J. and Poordad, F. (2015). Interferon-free, direct-acting antiviral therapy for chronic hepatitis C. *J. Viral Hepat.* **22**:861–870. DOI:https://doi.org/10.1111/jvh.12422.
5. AASLD/IDSA HCV Guidance Panel (2015). Hepatitis C guidance: AASLD-IDSA recommendations for testing, managing, and treating adults infected with hepatitis C virus. *Hepatology (Baltim., Md.)* **62**:932–954. DOI:https://doi.org/10.1002/hep.27950.
6. Denkinger, C.M. and Kessel, M. (2015). Diagnostics for hepatitis C: an urgent need for action. *Lancet. Glob. Health* **3**:e195. DOI:https://doi.org/10.1016/S2214-109X(15)70092-6.
7. WHO (2017). Global Hepatitis Report. Geneva, Available at: http://apps.who.int/iris/bitstream/10665/255016/1/9789241565455-eng.
8. Global Burden of Disease Study 2013 Collaborators (2015). Global, regional, and national incidence, prevalence, and years lived with disability for 301 acute and chronic diseases and injuries in 188 countries, 1990-2013: a systematic analysis for the Global Burden of Disease Study 2013. *Lancet* **386**:743–800. DOI:https://doi.org/10.1016/S0140-6736(15)60692-4.
9. Ivanova Reipold, E., Easterbrook, P., Trianni, A. et al. (2017). Optimising diagnosis of viraemic hepatitis C infection: the development of a target product profile. *BMC Infect. Dis.* **17**:707. DOI:https://doi.org/10.1186/s12879-017-2770-5.
10. WHO (2016). Global health strategy on viral hepatitis 2016-2021, Available at: https://apps.who.int/iris/bitstream/handle/10665/246177/WHO-HIV-2016.06-eng.pdf.
11. Tordrup, D., Hutin, Y., Stenberg, K. et al. (2019). Additional resource needs for viral hepatitis elimination through universal health coverage: projections in 67 low-income and middle-income countries, 2016-30. *Lancet. Glob. Health* **7**:e1180–e1188. DOI:https://doi.org/10.1016/S2214-109X(19)30272-4.
12. Ghany, M.G., Strader, D.B., Thomas, D.L. et al. (2009). Diagnosis, management, and treatment of hepatitis C: an update. *Hepatology (Baltim., Md.)* **49**:1335–1374. DOI:https://doi.org/10.1002/hep.22759.
13. CDC (2013). Testing for HCV infection: an update of guidance for clinicians and laboratorians, Available at: https://www.cdc.gov/mmwr/preview/mmwrhtml/mm6218a5.htm.
14. Yang, Y., Yang, M., Yuan, J. et al. (2020). Laboratory Diagnosis and Monitoring the Viral Shedding of SARS-CoV-2 Infection. *Innovation* **1**:10006. DOI:https://doi.org/10.1016/j.xinn.2020.100061.
15. Feld, J.J. and Ward, J.W. (2021). Key Elements on the Pathway to HCV Elimination: Lessons Learned From the AASLD HCV Special Interest Group 2020. *Hepatol. Commun.* **5**:911–922. DOI:https://doi.org/10.1002/hep4.1731.
16. Thomas, D.L. (2019). Global Elimination of Chronic Hepatitis. *N. Engl. J. Med.* **380**:2041–2050. DOI:https://doi.org/10.1056/NEJMra1810477.
17. JM, P. (1999). Diagnostic tests for hepatitis C. *J. Hepatol.* **31**:9.
18. Hanuka, N., Sikuler, E., Tovbin, D. et al. (2002). Hepatitis C virus infection in renal failure patients in the absence of anti-hepatitis C virus antibodies. *J. Viral Hepat.* **9**:141–145. DOI:https://doi.org/10.1046/j.1365-2893.2002.00332.x.
19. CDC (2016). Testing recommen- dations for hepatitis C virus infection. Available at: https://www.cdc.gov/hepatitis/hcv/guidelinesc.htm.
20. Pollock, K.G., McDonald, S.A., Gunson, R. et al. (2020). Real-world utility of HCV core antigen as an alternative to HCV RNA testing: Implications for viral load and genotype. *J. Viral Hepat.* **27**:996–1002. DOI:https://doi.org/10.1111/jvh.13337.
21. European Association for the Study of the Liver, Clinical Practice Guidelines Panel: Chair, EASL Governing Board Representative et al. (2020). EASL recommendations on treatment of hepatitis C: Final update of the series. *J. Hepatol.* **73**:1170–1218. DOI:https://doi.org/10.1016/j.jhep.2020.08.018.
22. Paul, J.C., Chulanov, V.P., Pimenov, N.N. et al. (2019). Streamlining the screening cascade for active Hepatitis C in Russia: A cost-effectiveness analysis. *PLoS One* **14**:e0219687.
23. van Tilborg, M., Al Marzooqi, S.H., Wong, W.W.L. et al. (2018). HCV core antigen as an alternative to HCV RNA testing in the era of direct-acting antivirals: retrospective screening and diagnostic cohort studies. *Lancet Gastroenterol. Hepatol.* **3**:856–864. DOI:https://doi.org/10.1016/S2468-1253(18)30271-1.
24. Fabrizi, F., Lunghi, G., Aucella, F. et al. (2005). Novel assay using total hepatitis C virus (HCV) core antigen quantification for diagnosis of HCV infection in dialysis patients. *J. Clin. Microbiol.* **43**:414–420. DOI:https://doi.org/10.1128/JCM.43.1.414-420.2005.
25. Aoyagi, K., Ohue, C., Iida, K. et al. (1999). Development of a simple and highly sensitive enzyme immunoassay for hepatitis C virus core antigen. *J. Clin. Microbiol.* **37**:1802–1808. DOI:https://doi.org/10.1128/JCM.37.6.1802-1808.1999.
26. Kaiser, T., Damerow, H.C., Tenckhoff, S. et al. (2008). Kinetics of hepatitis C viral RNA and HCV-antigen during dialysis sessions: evidence for differential viral load reduction on dialysis. *J. Med. Virol.* **80**:1195–1201. DOI:https://doi.org/10.1002/jmv.21190.
27. Schuttler, C.G., Thomas, C., Discher, T. et al. (2004). Variable ratio of hepatitis C virus RNA to viral core antigen in patient sera. *J. Clin. Microbiol.* **42**:1977–1981. DOI:https://doi.org/10.1128/JCM.42.5.1977-1981.2004.
28. Seme, K., Poljak, M., Babic, D.Z. et al. (2005). The role of core antigen detection in management of hepatitis C: a critical review. *J. Clin. Virol.* **32**:92–101. DOI:https://doi.org/10.1016/j.jcv.2004.10.005.
29. Bouvier-Alias, M., Patel, K., Dahari, H. et al. (2002). Clinical utility of total HCV core antigen quantification: a new indirect marker of HCV replication. *Hepatology (Baltim., Md.)* **36**:211–218. DOI:https://doi.org/10.1053/jhep.2002.34130.

www.the-innovation.org

30. Chen, D., Lu, Q., Song, N. et al. (2024). Nanostructured interface-engineered field-effect transistor biosensors for sensitive detection of serum miRNAs. *Innov. Mater.* **2**:100091. DOI:https://doi.org/10.59717/j.xinn-mater.2024.100091.
31. Rokuhara, A., Tanaka, E., Matsumoto, A. et al. (2003). Clinical evaluation of a new enzyme immunoassay for hepatitis B virus core-related antigen; a marker distinct from viral DNA for monitoring lamivudine treatment. *J. Viral Hepat.* **10**:324–330. DOI:https://doi.org/10.1046/j.1365-2893.2003.00437.x.
32. Tanaka, E., Ohue, C., Aoyagi, K. et al. (2000). Evaluation of a new enzyme immunoassay for hepatitis C virus (HCV) core antigen with clinical sensitivity approximating that of genomic amplification of HCV RNA. *Hepatology (Baltim., Md.)* **32**:388–393. DOI:https://doi.org/10.1053/jhep.2000.9112.
33. Lorenzo, J., Castro, A., Aguilera, A. et al. (2004). Total HCV core antigen assay. A new marker of HCV viremia and its application during treatment of chronic hepatitis C. *J. Virol. Methods* **120**:173–177. DOI:https://doi.org/10.1016/j.jviromet.2004.05.002.
34. Zanetti, A.R., Romanò, L., Brunetto, M. et al. (2003). Total HCV core antigen assay: a new marker of hepatitis C viremia for monitoring the progress of therapy. *J. Med. Virol.* **70**:27–30. DOI:https://doi.org/10.1002/jmv.10355.
35. Veillon, P., Payan, C., Picchio, G. et al. (2003). Comparative evaluation of the total hepatitis C virus core antigen, branched-DNA, and amplicor monitor assays in determining viremia for patients with chronic hepatitis C during interferon plus ribavirin combination therapy. *J. Clin. Microbiol.* **41**:3212–3220. DOI:https://doi.org/10.1128/JCM.41.7.3212-3220.2003.
36. Morota, K., Fujinami, R., Kinukawa, H. et al. (2009). A new sensitive and automated chemiluminescent microparticle immunoassay for quantitative determination of hepatitis C virus core antigen. *J. Virol. Methods* **157**:8–14. DOI:https://doi.org/10.1016/j.jviromet.2008.12.009.
37. Medici, M.C., Furlini, G., Rodella, A. et al. (2011). Hepatitis C virus core antigen: analytical performances, correlation with viremia and potential applications of a quantitative, automated immunoassay. *J. Clin. Virol.* **51**:264–269. DOI:https://doi.org/10.1016/j.jcv.2011.05.003.
38. Garbuglia, A.R., Monachetti, A., Galli, C. et al. (2014). HCV core antigen and HCV-RNA in HIV/HCV co-infected patients with different HCV genotypes. *BMC Infect. Dis.* **14**:222. DOI:https://doi.org/10.1186/1471-2334-14-222.
39. Ottiger, C., Gygli, N. and Huber, A.R. (2013). Detection limit of architect hepatitis C core antigen assay in correlation with HCV RNA, and renewed confirmation algorithm for reactive anti-HCV samples. *J. Clin. Virol.* **58**:535–540. DOI:https://doi.org/10.1016/j.jcv.2013.08.028.
40. Freiman, J.M., Tran, T.M., Schumacher, S.G. et al. (2016). Hepatitis C Core Antigen Testing for Diagnosis of Hepatitis C Virus Infection: A Systematic Review and Meta-analysis. *Ann. Intern. Med.* **165**:345–355. DOI:https://doi.org/10.7326/M16-0065.
41. Bertisch, B., Brezzi, M., Negro, F. et al. (2020). Very Low Hepatitis C Viral Loads in Treatment-naive Persons: Do They Compromise Hepatitis C Virus Antigen Testing? *Clin. Infect. Dis.* **70**:653–659.
42. Yeh, H.-W. and Ai, H.-W. (2019). Development and applications of bioluminescent and chemiluminescent reporters and biosensors. *Annu. Rev. Anal. Chem.* **12**:129–150.
43. Alsawaftah, N., Farooq, A., Dhou, S. et al. (2021). Bioluminescence imaging applications in cancer: A comprehensive review. *IEEE Rev. Biomed. Eng.* **14**:307–326.
44. Hosseini, S., Vázquez-Villegas, P., Rito-Palomares, M. et al. (2018). Advantages, disadvantages and modifications of conventional ELISA. In Enzyme-Linked Immunosorbent Assay (ELISA) from A to Z, pp. 67–115.
45. Barone, P.W., Parker, R.S. and Strano, M.S. (2005). In vivo fluorescence detection of glucose using a single-walled carbon nanotube optical sensor: design, fluorophore properties, advantages, and disadvantages. *Anal. Chem.* **77**:7556–7562.
46. Liu, B., Zhuang, J. and Wei, G. (2020). Recent advances in the design of colorimetric sensors for environmental monitoring. *Environ. Sci. Nano* **7**:2195–2213.
47. Gao, Y.-Z., Wang, Y., Ji, M. et al. (2023). A whole-cell hydrogen peroxide biosensor and its application in visual food analysis. *Innov. Life* **1**:100011. DOI:https://doi.org/10.59717/j.xinn-life.2023.100011.
48. Jin, W.-Y., Guo, J.-X., Tang, R. et al. (2024). In vivo detection of endogenous toxic phenolic compounds of intestine. *J. Hazard. Mater.* **478**:135526. DOI:https://doi.org/10.1016/j.jhazmat.2024.135526.
49. Carriba, P., Navarro, G., Ciruela, F. et al. (2008). Detection of heteromerization of more than two proteins by sequential BRET-FRET. *Nat. Methods* **5**:727–733.
50. Pfleger, K.D.G. and Eidne, K.A. (2006). Illuminating insights into protein-protein interactions using bioluminescence resonance energy transfer (BRET). *Nat. Methods* **3**:165–174.
51. Smirnova, D.V. and Ugarova, N.N. (2017). Firefly luciferase-based fusion proteins and their applications in bioanalysis. *Photochem. Photobiol.* **93**:436–447.
52. Hall, M.P., Kincaid, V.A., Jost, E.A. et al. (2021). Toward a point-of-need bioluminescence-based immunoassay utilizing a complete shelf-stable reagent. *Anal. Chem.* **93**:5177–5184.
53. Elledge, S.K., Zhou, X.X., Byrnes, J.R. et al. (2021). Engineering luminescent biosensors for point-of-care SARS-CoV-2 antibody detection. *Nat. Biotechnol.* **39**:928–935.
54. Ravalin, M., Roh, H., Suryawanshi, R. et al. (2022). A single-component luminescent biosensor for the SARS-CoV-2 spike protein. *J. Am. Chem. Soc.* **144**:13663–13672.
55. Hemmati, R., Hosseinkhani, S., Sajedi, R.H. et al. (2015). Luciferin-regenerating enzyme mediates firefly luciferase activation through direct effects of D-cysteine on luciferase structure and activity. *Photochem. Photobiol.* **91**:828–836.
56. Fraga, H., Fernandes, D., Fontes, R. et al. (2005). Coenzyme A affects firefly luciferase luminescence because it acts as a substrate and not as an allosteric effector. *FEBS J.* **272**:5206–5216.
57. Huang, T., Xu, H., Wang, H. et al. (2023). Artificial intelligence for medicine: Progress, challenges, and perspectives. *Innov. Med.* **1**:100030. DOI:https://doi.org/10.59717/j.xinn-med.2023.100030.
58. WHO (2021). Consolidated guidelines on HIV prevention, testing, treatment, service delivert and monitoring, Available at: https://www.who.int/publications/i/item/9789240031593.
59. WHO (2021). Updated recommendations on HIV Prevention, infant diagnosis, antiretroviral initiation and monitoring, Available at: https://iris.who.int/bitstream/handle/10665/340190/9789240022232-eng.pdf?sequence=1.
60. Pawlotsky, J.-M., Negro, F., Aghemo, A. et al. (2018). EASL recommendations on treatment of hepatitis C 2018. *J. Hepatol.* **69**:461–511.
61. Grebely, J., Lamoury, F.M.J., Hajarizadeh, B. et al. (2017). Evaluation of the Xpert HCV Viral Load point-of-care assay from venepuncture-collected and finger-stick capillary whole-blood samples: a cohort study. *Lancet Gastroenterol. Hepatol.* **2**:514–520.
62. WHO (2021). WHO public reports for in-vitro diagnostics, Available at: https://extranet.who.int/pqweb/vitro-diagnostics/prequalification-reports/whopr.
63. Jung, Y.K., Park, H.G. and Kim, J.-M. (2006). Polydiacetylene (PDA)-based colorimetric detection of biotin–streptavidin interactions. *Biosens. Bioelectron.* **21**:1536–1544.
64. Serrano-Plana, J., Rumo, C., Rebelein, J.G. et al. (2020). Enantioselective Hydroxylation of Benzylic C (sp3)–H Bonds by an Artificial Iron Hydroxylase Based on the Biotin–Streptavidin Technology. *J. Am. Chem. Soc.* **142**:10617–10623.
65. Warkad, S.D., Song, K.S., Pal, D. et al. (2019). Developments in the HCV Screening Technologies Based on the Detection of Antigens and Antibodies. *Sensors (Basel)* **19**:4257. DOI:https://doi.org/10.3390/s19194257.
66. Wang, C., Zhang, L. and Shen, X. (2013). Development of a nucleic acid lateral flow strip for detection of hepatitis C virus (HCV) core antigen. *Nucleosides Nucleotides Nucleic Acids* **32**:59–68.
67. Frimpong, J.A., D'Aunno, T., Perlman, D.C. et al. (2016). On-site bundled rapid HIV/HCV testing in substance use disorder treatment programs: study protocol for a hybrid design randomized controlled trial. *Trials* **17**:117. DOI:https://doi.org/10.1186/s13063-016-1225-4.
68. Price, C.P. (2001). Point-of-care testing. Impact on medical outcomes. *Clin. Lab. Med.* **21**:285–303.
69. APHL (2021). Identifying High-Priority Diagnostic Approaches for Advancing Hepatitis C Elimination in the US, Available at: https://www.aphl.org/programs/infectious_disease/Pages/Hepatitis-C-Virus-Diagnostic-Meeting.aspx.

The Innovation, Volume *6*

Supplemental Information

A fully automated, ultrasensitive luminescence cascade sensor to address hepatitis C diagnostic disparity

Sungwan Kim, Adharsh Chellappaa, Juhyeon Chun, Jaebaek Lee, Joseph M. Hardie, Manoj K. Kanakasabapathy, Hemanth Kandula, Prudhvi Thirumalaraju, Gregory P. Fricker, Jenna Gustafson, Raymond T. Chung, Jorge Mera, and Hadi Shafiee

Table of Contents

Figure S1. ELISA of dAb-biotin.
Figure S2. Optimization of air volume for sample, washing buffer, and working solution injection.
Figure S3. Effect of washing buffer for the generation of bioluminescence.
Figure S4. Blocking of the channel and chamber of microfluidic cartridge.
Figure S5. Instruction sheet used for sample testing by untrained users.
Figure S6. Dose-response curve of bioluminescence intensity (RLU) from the automated assay in response to varying concentrations of serially diluted HCV-spiked samples.
Figure S7. Calculation of Cohen’s κ coefficient.

Table S1. Estimated materials costs for the automated assay.
Table S2. The results obtained with the qualitative enzyme cascade-based bioluminescence system using HCV-spiked plasma samples.
Table S3. The results obtained with the qualitative enzyme cascade-based bioluminescence system using HCV-infected Cherokee Nation patient samples.
Table S4. Qualitative enzyme cascade-based bioluminescence system assessment results using HCV-spiked plasma samples.
Table S5. Qualitative conventional bioluminescence system assessment results using HCV-spiked plasma samples.
Table S6. Qualitative ELISA assessment results using HCV-spiked plasma samples.
Table S7. Qualitative qPCR assessment results using HCV-spiked plasma samples.
Table S8. Head-to-head comparison of enzyme cascade-based assay with conventional bioluminescence system, ELISA, and qPCR.

A

HCV	1	2	3	4	5	6
Core antigen	+	+	-	-	+	+
dAb-biotin	+	+	+	+	-	-
Streptavidin-HRP	+	-	+	-	+	-
Anti-IgG-HRP	-	+	-	+	-	+

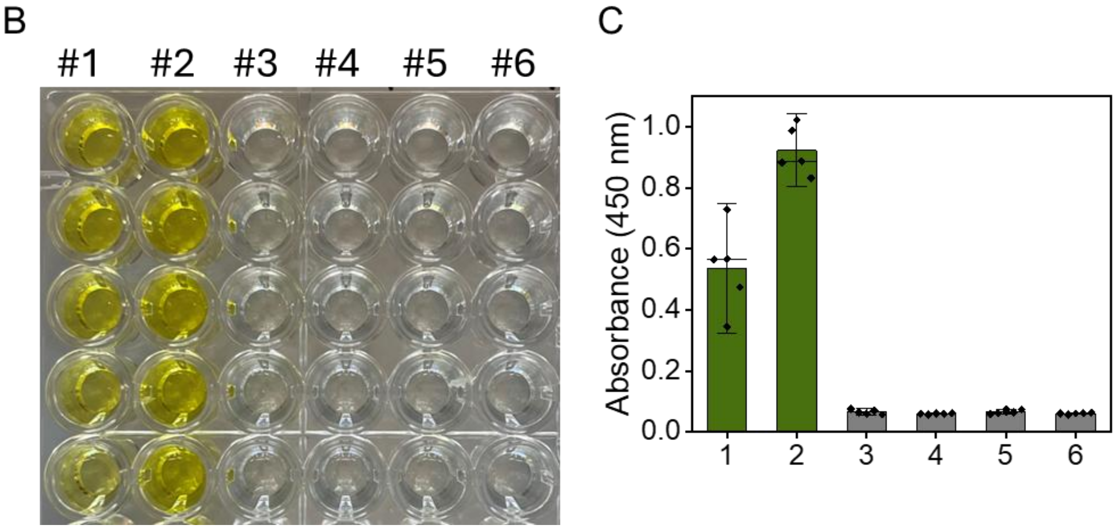

Figure S1. ELISA of dAb-biotin. (**A**) Experimental condition of ELISA. (**B**) Snapshot of the ELISA results. (**C**) ELISA analysis of dAb-biotin with antibodies targeting HCV. Bars represent mean ± SD with n = 5 technical replicates.

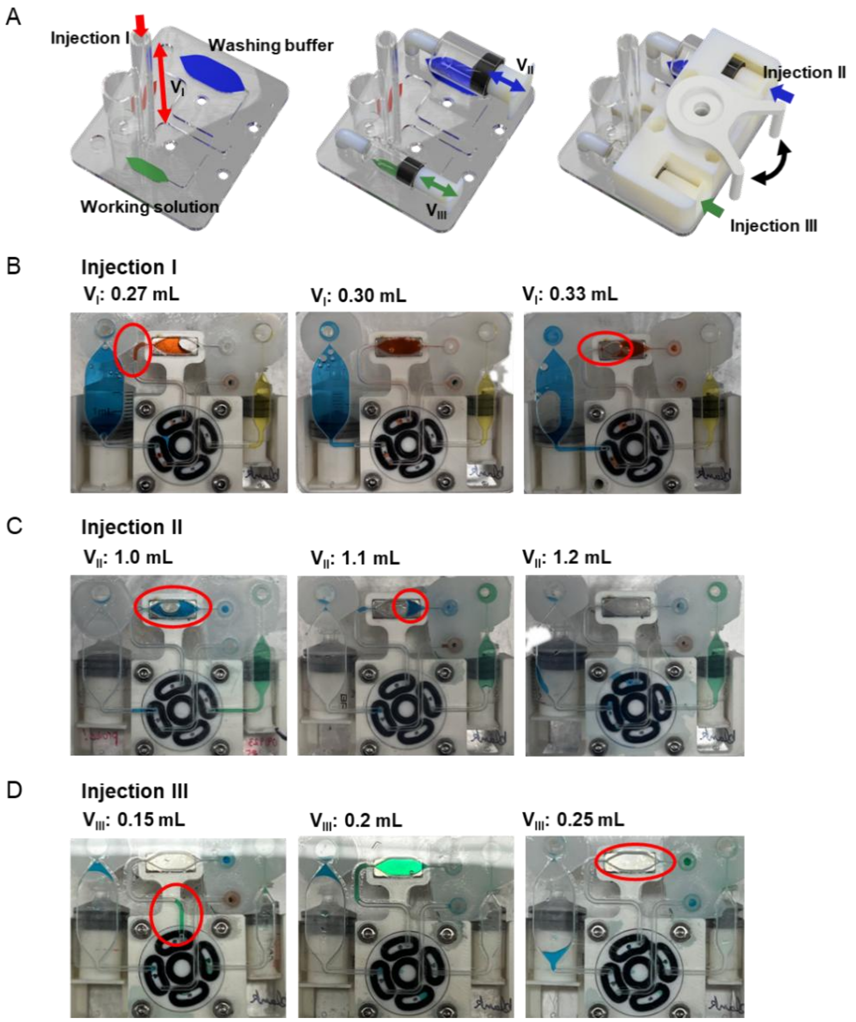

Figure S2. Optimization of air volume for sample, washing buffer, and working solution injection. (**A**) Microfluidic cartridge image. Optimization of air volume for (**B**) sample, (**C**) washing buffer, and (**D**) working solution using food dye. The colors of food dyes, red, blue, and green, correspond to sample, washing buffer, and working solution, respectively. Red circles indicate the inability of the solution to reach the appropriate position.

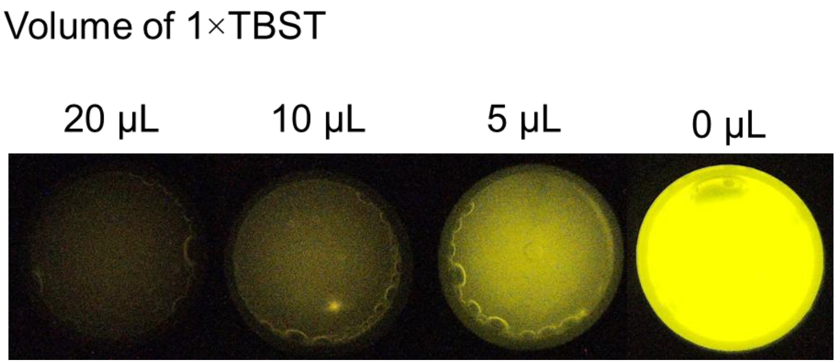

Figure S3. Effect of washing buffer for the generation of bioluminescence. The ST-GAL (10 ng) was incubated with different volume of 1×TBST (washing buffer) 0, 5, 10, 20 μL and working solution (80 μL) to generate bioluminescence.

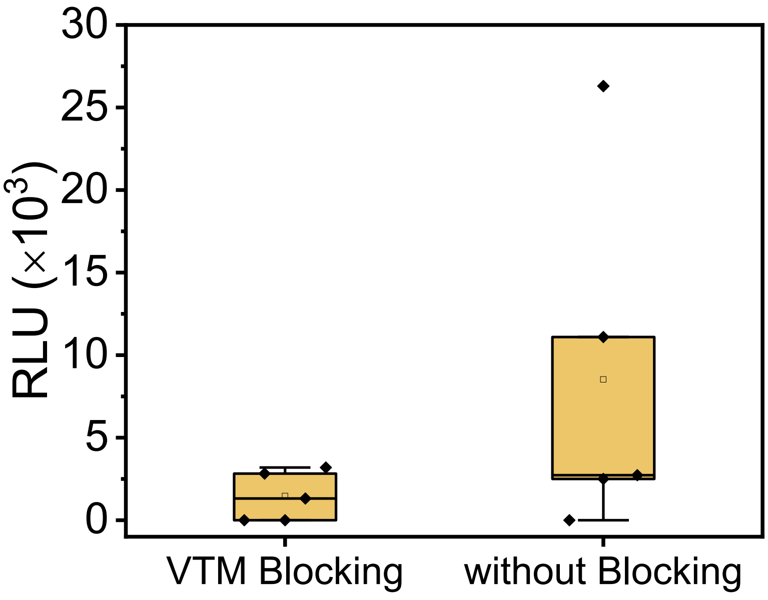

Figure S4. Blocking of the channel and chamber of microfluidic cartridge. The automated assay was performed under blank condition (using cAb-MgBeads and dAb-GAL without target protein) with or without treatment of microfluidic cartridges using VTM. Bars represent mean ± SD with n = 5 technical replicates.

User Instruction Sheet

1- Please follow these step-by-step instructions to test your sample using the extraction buffer tube (bag-1) and the detection module (bag-2). All necessary reagents are included in the provided materials.

2- Step-by-step protocol:

**Step#1**	Introduce the test sample into the extraction buffer tube and swirl it for 30 seconds.<br><br>Gently squeeze the tube to dispense the entire solution into the sample injection syringe.	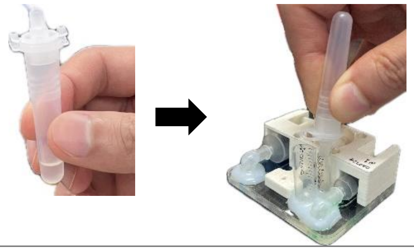
**Step#2**	Push the solution with injection bar.	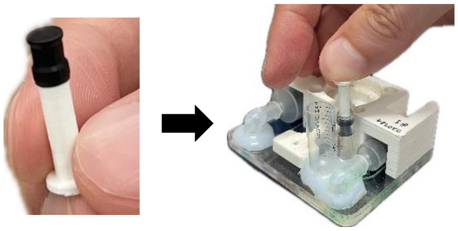
**Step#3**	Attach a disposable cartridge to the detection module and close the lid.	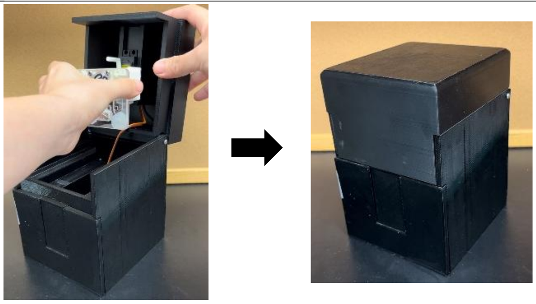
**Step#4**	Press the “Start” button in the smartphone app and wait for the assay result to be displayed.	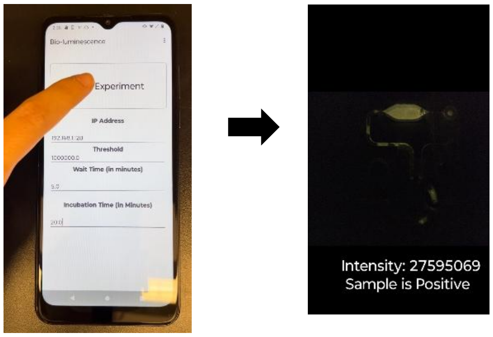

3- Dispose of the assay chamber and keep the detection module for the next experiment.

Figure S5. Instruction sheet used for sample testing by untrained users.

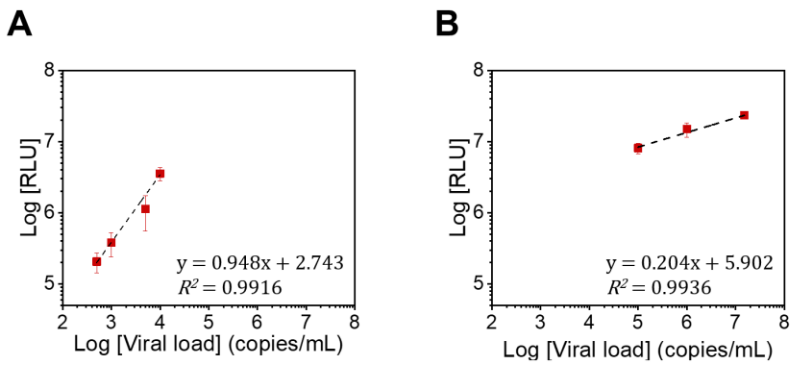

Figure S6. Dose-response curve of bioluminescence intensity (RLU) from the automated assay in response to varying concentrations of serially diluted HCV-spiked samples. (**A**) 500 to 10^4 copies/mL and (**B**) 10^5 to 10^7 copies/mL. The samples were mixed with assay materials and processed according to the protocol described in the automated assay protocol of the enzyme cascade-based bioluminescence system. Data are mean ± SD with n = 5 technical replicates.

Positive: 37
Negative: 34
False Positive: 4
False Negative: 0

A (expected True Positive): $\frac{37*71}{71} = 37$

D (expected True Negative): $\frac{34+71}{71} = 34$

B (False Positive): $\frac{4*71}{71} = 4$

C (False Negative): $\frac{0*71}{71} = 0$

$$P_0 = \frac{Number\ of\ agreements}{Total\ number\ of\ cases} = \frac{67}{71} = 0.944$$

$$P_e = \frac{(A+B)*(A+C)+(D+B)*(D+C)}{n^2}$$
$$= \frac{(37+4)*(37+0)+(34+4)(34+0)}{71^2} = 0.557$$

$$\kappa = \frac{P_0 - P_e}{1 - P_e} = \frac{0.944 - 0.557}{1 - 0.557} = 0.875$$

Figure S7. Calculation of Cohen's κ coefficient.

Table S1. Estimated materials costs for the automated assay.

Item	*Cost (USD)*
Microfluidic Cartridge	
PMMA/DSA	0.001
PLA	0.119
Syringe	0.06
O-rings	0.05
cAb-MgBeads (cAb and MgBeads)	0.54
cAb	0.44
MgBeads	0.10
dAb-GAL (dAb, Site specific biotinylation and ST-GAL)	0.40
dAb	0.11
Site specific biotinylation	0.26
ST-GAL	0.03
Washing buffer	0.001
Working solution (LUGAL, Fluc, ATP, Co-enzyme A and Additives)	0.73
LUGAL	0.10
Fluc	0.50
ATP	0.11
Co-enzyme A	0.01
Additives (TCEP, EDTA, $MgSO_4 \cdot 7H_2O$ and BSA)	0.01
Bolts & Nuts	0.40
Total	2.301
Detection Module	
PLA	2.97
Arduino Nano	2.05
Raspberry Pi 4	36.38
CMOS sensor	24.99
Servo Motor	1.80
Battery	7.00
U Shape Type C Male to Female 40 Gbps Connector	1.92
Type A Male to USB 3.1 Type C Male Up Opposite U Shape Back Angled 90 Degree Charge Adapter	7.78
Jumper wires	0.175
Ball bearing 6802 RS	1.30
Bolts & Nuts	0.47
Total	86.84

Table S2. The results obtained with the qualitative enzyme cascade-based bioluminescence system using HCV-spiked plasma samples (True Positive: viral load ≥ 768 copies/mL, True Negative: viral load < 768 copies/mL, and Positive, False Positive and Negative: The results obtained by our qualitative enzyme cascade-based bioluminescence assay).

Sample #	Viral Load (copies/mL) (qPCR)	Qualitative PCR	Qualitative enzyme cascade-based bioluminescence system
1	15,000,000	True Positive	Positive
2	15,000,000	True Positive	Positive
3	15,000,000	True Positive	Positive
4	15,000,000	True Positive	Positive
5	15,000,000	True Positive	Positive
6	1,000,000	True Positive	Positive
7	1,000,000	True Positive	Positive
8	1,000,000	True Positive	Positive
9	1,000,000	True Positive	Positive
10	1,000,000	True Positive	Positive
11	100,000	True Positive	Positive
12	100,000	True Positive	Positive
13	100,000	True Positive	Positive
14	100,000	True Positive	Positive
15	100,000	True Positive	Positive
16	10,000	True Positive	Positive
17	10,000	True Positive	Positive
18	10,000	True Positive	Positive
19	10,000	True Positive	Positive
20	10,000	True Positive	Positive
21	5,000	True Positive	Positive
22	5,000	True Positive	Positive
23	5,000	True Positive	Positive
24	5,000	True Positive	Positive
25	5,000	True Positive	Positive
26	1,000	True Positive	Positive
27	1,000	True Positive	Positive
28	1,000	True Positive	Positive
29	1,000	True Positive	Positive
30	1,000	True Positive	Positive

31	500	True Negative	Negative
32	500	True Negative	Negative
33	500	True Negative	False Positive
34	500	True Negative	False Positive
35	500	True Negative	False Positive
36	350	True Negative	Negative
37	250	True Negative	Negative
38	150	True Negative	Negative
39	100	True Negative	Negative
40	80	True Negative	Negative
41	50	True Negative	Negative
42	30	True Negative	Negative
43	10	True Negative	False Positive
44	0	True Negative	Negative
45	0	True Negative	Negative
46	0	True Negative	Negative
47	0	True Negative	Negative
48	0	True Negative	Negative
49	0	True Negative	Negative
50	0	True Negative	Negative
51	0	True Negative	Negative
52	0	True Negative	Negative
53	0	True Negative	Negative
54	0	True Negative	Negative
55	0	True Negative	Negative
56	0	True Negative	Negative
57	0	True Negative	Negative
58	0	True Negative	Negative
59	0	True Negative	Negative
60	0	True Negative	Negative
61	0	True Negative	Negative
62	0	True Negative	Negative
63	0	True Negative	Negative
64	0	True Negative	Negative
65	0	True Negative	Negative
66	0	True Negative	Negative

67	0	True Negative	Negative
68	0	True Negative	Negative
69	0	True Negative	Negative
70	0	True Negative	Negative
71	0	True Negative	Negative
72	0	True Negative	Negative
73	0	True Negative	Negative
74	0	True Negative	Negative
75	0	True Negative	Negative
76	0	True Negative	Negative
77	0	True Negative	Negative
78	0	True Negative	Negative
79	0	True Negative	Negative
80	0	True Negative	Negative
81	0	True Negative	Negative
82	0	True Negative	Negative
83	0	True Negative	Negative
84	0	True Negative	Negative
85	0	True Negative	Negative
86	0	True Negative	Negative
87	0	True Negative	Negative
88	0	True Negative	Negative
89	0	True Negative	Negative
90	0	True Negative	Negative
91	0	True Negative	Negative
92	0	True Negative	Negative

Table S3. The results obtained with the qualitative enzyme cascade-based bioluminescence system using HCV-infected Cherokee Nation patient samples (True Positive: viral load ≥ 768 copies/mL, True Negative: viral load < 768 copies/mL, and Positive, False Positive, Negative and False Negative: The results obtained by our qualitative enzyme cascade-based bioluminescence assay).

Patient ID	HCV (copies/mL)	Qualitative PCR	Qualitative enzyme cascade-based bioluminescence system
HAR016	2,000	True Positive	Positive
HAR034	2,000	True Positive	False Negative
HAR032	20,000	True Positive	False Negative
HAR039	75,000	True Positive	Positive
HAR007	100,000	True Positive	Positive
HAR005	150,000	True Positive	Positive
HAR028	150,000	True Positive	Positive
HAR033	150,000	True Positive	Positive
HAR035	150,000	True Positive	Positive
HAR009	178,996	True Positive	Positive
HAR036	300,000	True Positive	Positive
HAR020	326,338	True Positive	Positive
HAR041	350,000	True Positive	Positive
HAR031	500,000	True Positive	Positive
HAR006	604,320	True Positive	Positive
HAR023	1,000,000	True Positive	Positive
HAR030	1,000,000	True Positive	Positive
HAR038	1,000,000	True Positive	Positive
HAR019	1,742,638	True Positive	Positive
HAR040	1,750,000	True Positive	Positive
HAR022	2,000,000	True Positive	Positive
HAR026	2,500,000	True Positive	Positive
HAR037	3,000,000	True Positive	Positive
HAR003	3,140,352	True Positive	Positive
HAR025	5,000,000	True Positive	Positive
HAR042	5,000,000	True Positive	Positive
HAR011	6,219,884	True Positive	Positive
HAR015	8,000,000	True Positive	Positive
HAR043	9,500,000	True Positive	Positive
HAR021	10,000,000	True Positive	Positive
HAR002	10,000,000	True Positive	Positive
HAR017	13,000,000	True Positive	Positive
HAR012	14,000,000	True Positive	Positive
HAR014	15,000,000	True Positive	Positive
HAR044	15,000,000	True Positive	Positive
HAR010	18,270,496	True Positive	Positive
HAR027	19,000,000	True Positive	Positive

Table S3. Continued.

Patient ID	HCV (copies/mL)	Qualitative PCR	Qualitative enzyme cascade-based bioluminescence system
HAR029	15	True Negative	Negative
HAR024	500	True Negative	Negative
HAR013	700	True Negative	False Positive
HAR018	718	True Negative	Negative
N1	114.25	True Negative	Negative
N2	213.5	True Negative	Negative
N3	312.75	True Negative	Negative
N4	412	True Negative	Negative
N5	511.25	True Negative	Negative
N6	50.15	True Negative	Negative
N7	85.3	True Negative	Negative
N8	120.45	True Negative	Negative
N9	155.6	True Negative	Negative
N10	190.75	True Negative	Negative
N11	225.9	True Negative	Negative
N12	261.05	True Negative	Negative
N13	296.2	True Negative	Negative
N14	331.35	True Negative	Negative
N15	366.5	True Negative	Negative
N16	401.65	True Negative	Negative
N17	436.8	True Negative	Negative
N18	471.95	True Negative	Negative
N19	507.1	True Negative	Negative
N20	542.25	True Negative	Negative
N21	39.25	True Negative	Negative
N22	63.5	True Negative	Negative
N23	87.75	True Negative	Negative
N24	112	True Negative	Negative
N25	136.25	True Negative	Negative
N26	160.5	True Negative	Negative
N27	184.75	True Negative	Negative
N28	209	True Negative	Negative
N29	510	True Negative	Negative
N30	575	True Negative	Negative

Table S4. Qualitative enzyme cascade-based bioluminescence system assessment results using HCV-spiked plasma samples (Positive and Negative: qualitative results obtained by the enzyme cascade-based bioluminescence system based on the viral load threshold of 768 copies/mL).

Positive			Negative		
**Sample #**	**Results**	**Viral Load (copies/mL) (qPCR)**	**Sample #**	**Results**	**Viral Load (copies/mL) (qPCR)**
1	Positive	15,000,000	24	Negative	700
2	Positive	13,000,000	25	Negative	500
3	Positive	1,000,000	26	Negative	350
4	Positive	900,000	27	Negative	250
5	Positive	800,000	28	Negative	150
6	Positive	700,000	29	Negative	100
7	Positive	650,000	30	Negative	80
8	Positive	500,000	31	Negative	0
9	Positive	350,000	32	Negative	0
10	Positive	250,000	33	Negative	0
11	Positive	150,000	34	Negative	0
12	Positive	100,000	35	Negative	0
13	Positive	80,000	36	Negative	0
14	Positive	60,000	37	Negative	0
15	Positive	40,000	38	Negative	0
16	Positive	20,000	39	Negative	0
17	Positive	10,000	40	Negative	0
18	Positive	7,500	41	Negative	0
19	Positive	5,000	42	Negative	0
20	Positive	2,000	43	Negative	0
21	Positive	1,500	44	Negative	0
22	Positive	1,000	45	Negative	0
23	Positive	900	46	Negative	0
-	-	-	47	Negative	0
-	-	-	48	Negative	0
-	-	-	49	Negative	0
-	-	-	50	Negative	0

Table S5. Qualitative conventional bioluminescence system assessment results using HCV-spiked plasma samples (Positive, False Negative, and Negative: qualitative results obtained by the qualitative conventional bioluminescence system based on the viral load threshold of 768 copies/mL).

Positive			Negative		
**Sample #**	**Results**	**Viral Load (copies/mL) (qPCR)**	**Sample #**	**Results**	**Viral Load (copies/mL) (qPCR)**
1	Positive	15,000,000	24	Negative	700
2	Positive	13,000,000	25	Negative	500
3	Positive	1,000,000	26	Negative	350
4	Positive	900,000	27	Negative	250
5	Positive	800,000	28	Negative	150
6	Positive	700,000	29	Negative	100
7	Positive	650,000	30	Negative	80
8	Positive	500,000	31	Negative	0
9	Positive	350,000	32	Negative	0
10	Positive	250,000	33	Negative	0
11	Positive	150,000	34	Negative	0
12	Positive	100,000	35	Negative	0
13	Positive	80,000	36	Negative	0
14	Positive	60,000	37	Negative	0
15	False Negative	40,000	38	Negative	0
16	False Negative	20,000	39	Negative	0
17	False Negative	10,000	40	Negative	0
18	False Negative	7,500	41	Negative	0
19	False Negative	5,000	42	Negative	0
20	False Negative	2,000	43	Negative	0
21	False Negative	1,500	44	Negative	0
22	False Negative	1,000	45	Negative	0
23	False Negative	900	46	Negative	0
-	-	-	47	Negative	0
-	-	-	48	Negative	0
-	-	-	49	Negative	0
-	-	-	50	Negative	0

Table S6. Qualitative ELISA assessment results using HCV-spiked plasma samples (Positive, False Negative, and Negative: qualitative results obtained by the ELISA based on the viral load threshold of 768 copies/mL).

Positive			Negative		
**Sample #**	**Results**	**Viral Load (copies/mL) (qPCR)**	**Sample #**	**Results**	**Viral Load (copies/mL) (qPCR)**
1	Positive	15,000,000	24	Negative	700
2	Positive	13,000,000	25	Negative	500
3	Positive	1,000,000	26	Negative	350
4	Positive	900,000	27	Negative	250
5	Positive	800,000	28	Negative	150
6	Positive	700,000	29	Negative	100
7	Positive	650,000	30	Negative	80
8	Positive	500,000	31	Negative	0
9	Positive	350,000	32	Negative	0
10	Positive	250,000	33	Negative	0
11	Positive	150,000	34	Negative	0
12	Positive	100,000	35	Negative	0
13	Positive	80,000	36	Negative	0
14	Positive	60,000	37	Negative	0
15	Positive	40,000	38	Negative	0
16	Positive	20,000	39	Negative	0
17	Positive	10,000	40	Negative	0
18	False Negative	7,500	41	Negative	0
19	False Negative	5,000	42	Negative	0
20	False Negative	2,000	43	Negative	0
21	False Negative	1,500	44	Negative	0
22	False Negative	1,000	45	Negative	0
23	False Negative	900	46	Negative	0
-	-	-	47	Negative	0
-	-	-	48	Negative	0
-	-	-	49	Negative	0
-	-	-	50	Negative	0

Table S7. Qualitative qPCR assessment results using HCV-spiked plasma samples (Positive and Negative: qualitative results obtained by the qPCR based on the viral load threshold of 768 copies/mL).

Positive			Negative		
Sample #	Results	Viral Load (copies/mL) (qPCR)	Sample #	Results	Viral Load (copies/mL) (qPCR)
1	Positive	15,000,000	24	Negative	700
2	Positive	13,000,000	25	Negative	500
3	Positive	1,000,000	26	Negative	350
4	Positive	900,000	27	Negative	250
5	Positive	800,000	28	Negative	150
6	Positive	700,000	29	Negative	100
7	Positive	650,000	30	Negative	80
8	Positive	500,000	31	Negative	0
9	Positive	350,000	32	Negative	0
10	Positive	250,000	33	Negative	0
11	Positive	150,000	34	Negative	0
12	Positive	100,000	35	Negative	0
13	Positive	80,000	36	Negative	0
14	Positive	60,000	37	Negative	0
15	Positive	40,000	38	Negative	0
16	Positive	20,000	39	Negative	0
17	Positive	10,000	40	Negative	0
18	Positive	7,500	41	Negative	0
19	Positive	5,000	42	Negative	0
20	Positive	2,000	43	Negative	0
21	Positive	1,500	44	Negative	0
22	Positive	1,000	45	Negative	0
23	Positive	900	46	Negative	0
-	-	-	47	Negative	0
-	-	-	48	Negative	0
-	-	-	49	Negative	0
-	-	-	50	Negative	0

Table S8. Head-to-head comparison of enzyme cascade-based assay with conventional bioluminescence system, ELISA, and qPCR.

	Enzyme cascade-based bioluminescence system	Conventional bioluminescence system	ELISA	**qPCR**
Specificity	**100%**	**100%**	**100%**	**100%**
Sensitivity	**100%**	**61%**	**74%**	**100%**
Accuracy	**100%**	**78%**	**84%**	**100%**
